# Behavioural and neuroplastic effects of a double-blind randomised controlled balance exercise trial in people with Parkinson’s disease

**DOI:** 10.1038/s41531-021-00269-5

**Published:** 2022-01-21

**Authors:** Malin Freidle, Hanna Johansson, Urban Ekman, Alexander V. Lebedev, Ellika Schalling, William H. Thompson, Per Svenningsson, Martin Lövdén, Alonso Abney, Franziska Albrecht, Hanna Steurer, Breiffni Leavy, Staffan Holmin, Maria Hagströmer, Erika Franzén

**Affiliations:** 1 Department of Neurobiology, Care Sciences and Society, Division of Physiotherapy, Karolinska Institutet, Stockholm, Sweden; 2 Women’s Health and Allied Health Professionals Theme, Medical Unit Occupational Therapy & Physiotherapy, Karolinska University Hospital, Stockholm, Sweden; 3 Department of Neurobiology, Care Sciences and Society, Division of Clinical Geriatrics, Karolinska Institutet, Stockholm, Sweden; 4 Women’s Health and Allied Health Professionals Theme, Medical Unit Medical Psychology, Karolinska University Hospital, Stockholm, Sweden; 5 Department of Clinical Neuroscience, Division of Neuro, Karolinska Institutet, Stockholm, Sweden; 6 Department of Neurobiology, Care Sciences and Society, Aging Research Center, Karolinska Institutet, Stockholm, Sweden; 7 Department of Public Health and Caring Sciences, Uppsala University, Uppsala, Sweden; 8 Speech-Language Pathology, Uppsala University Hospital, Uppsala, Sweden; 9 Department of Clinical Science, Intervention and Technology—CLINTEC, Division of Speech and Language Pathology, Karolinska Institutet, Stockholm, Sweden; 10 Department of Psychology, University of Gothenburg, Gothenburg, Sweden; 11 R&D Unit, Stockholms Sjukhem, Stockholm, Sweden; 12 Academic Primary Health Care Centre, Region Stockholm, Stockholm, Sweden

**Keywords:** Parkinson's disease, Motor control, Randomized controlled trials, Magnetic resonance imaging

## Abstract

Balance dysfunction is a disabling symptom in people with Parkinson’s disease (PD). Evidence suggests that exercise can improve balance performance and induce neuroplastic effects. We hypothesised that a 10-week balance intervention (HiBalance) would improve balance, other motor and cognitive symptoms, and alter task-evoked brain activity in people with PD. We performed a double-blind randomised controlled trial (RCT) where 95 participants with PD were randomised to either HiBalance (*n* = 48) or a control group (*n* = 47). We found no significant group by time effect on balance performance (*b* = 0.4 95% CI [−1, 1.9], *p* = 0.57) or on our secondary outcomes, including the measures of task-evoked brain activity. The findings of this well-powered, double-blind RCT contrast previous studies of the HiBalance programme but are congruent with other double-blind RCTs of physical exercise in PD. The divergent results raise important questions on how to optimise physical exercise interventions for people with PD.

Preregistration clinicaltrials.gov: NCT03213873.

## Introduction

Worldwide over 6.1 million people live with Parkinson’s disease (PD), the fastest-growing neurological disorder to date^1^. Although primarily associated with a dopamine deficiency in the basal ganglia, PD also affects several frontal brain regions^2^. The complex plethora of PD symptoms include motor dysfunctions such as bradykinesia, rest tremor, rigidity, balance and gait impairments as well as deficits in motor learning^3,4^. In addition, impairments in executive function and speech are common and negatively impact daily living, quality of life and further add layers of complexity to treatment^5,6^. Physical exercise can ameliorate PD symptoms and serve as a valuable complement to pharmacological interventions for people with PD^7–9^.

Balance and gait impairments are some of the most debilitating symptoms in people with PD even after optimal medical management^10,11^. Our research group has previously developed a framework of highly challenging balance exercises for people with PD: the HiBalance programme^12,13^. The programme was developed based on the principles that physical exercise need to be performed near or at the limit of one’s capacity, specific to the impaired functions and performed in a progressive and varied manner^13–15^. Dual tasks (i.e., a secondary motor or cognitive task) are used as means of increasing the complexity and further challenge each individual. We have previously evaluated the HiBalance programme both in research settings^12^, and in real-world care settings^16^ with encouraging effects on balance and gait ability.

Recently synthesised evidence suggests that physical exercise can induce neuroplastic changes at molecular, structural and functional levels^17^. Although the precise mechanisms of exercise-induced neuroplasticity are still unclear, increased levels of brain-derived neurotrophic factor (BDNF) have been suggested to promote both neuroprotection and neuroregeneration^18^. Whether behavioural improvements, such as balance and gait seen in our earlier studies of the HiBalance programme, are associated with changes in markers of neural plasticity remains unanswered. For simplicity, we will further refer to measures of physical and cognitive functions as well as other health-related outcomes as behavioural outcomes.

To this end, the EXercise in PArkinson’s disease and Neuroplasticity (EXPANd) trial had two main aims. First, to evaluate the effect of the HiBalance programme on a range of behavioural outcomes including balance, gait, and executive function. Second, to investigate the relationship between changes in balance, gait, and executive function with changes in task-evoked brain activity measured with functional magnetic resonance imaging (fMRI) and changes in BDNF. The HiBalance programme was compared to an active control group (a speech and communication intervention called HiCommunication). We hypothesised greater improvements in balance performance (primary outcome), gait and executive function for the HiBalance group than for the active control group. We also hypothesised that the correlations between change (pre to post assessment) in balance performance, gait, and executive function, with change in task-evoked fMRI and BDNF, would be larger for the HiBalance group than for the active control group. The analysis plan was preregistered on the Open Science Framework (https://osf.io/s952g/), which includes hypotheses on other secondary outcomes.

## Results

### Study flow and baseline data

**Fig. 1:**
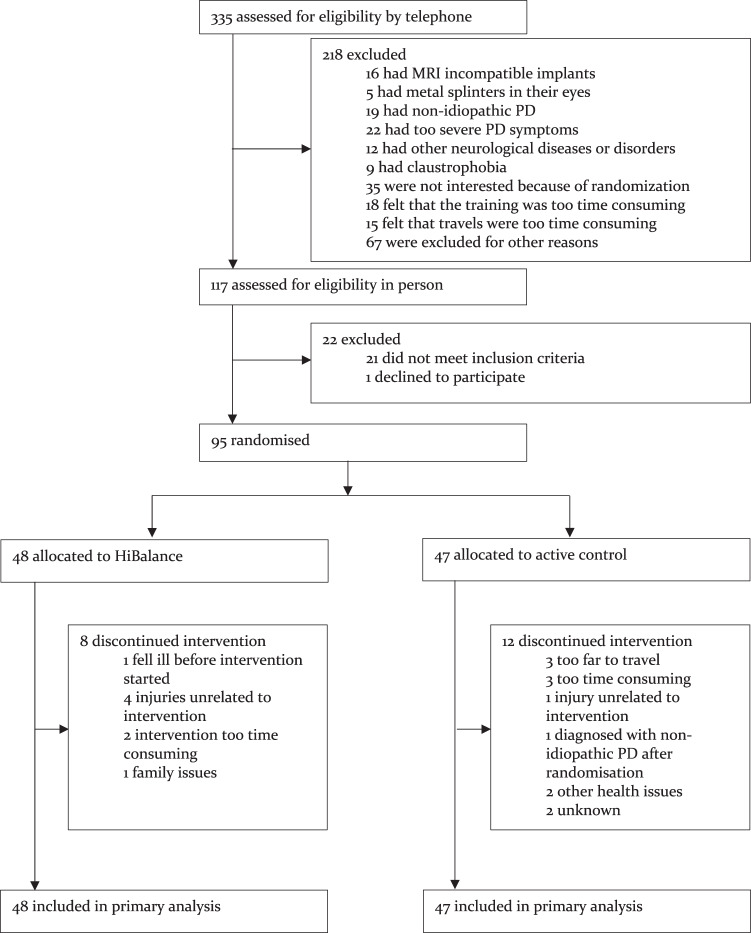
Trial flowchart. Details of the recruitment and study flow.

Inclusion occurred between 15 January 2018 and 9 September 2019. We screened 335 individuals that resulted in the inclusion of 95 participants who were randomly assigned to either the HiBalance group (*n* = 48) or the active control group (*n* = 47). See Table 1 for the baseline characteristics of the intention to treat sample and Fig. 1 for details of the recruitment process and attrition. Twenty patients discontinued their allocated exercise programme. This resulted in a total attrition rate of 21%: 17% for the HiBalance group and 26% for the active control group.

**Table 1:** Baseline characteristics of the intention-to-treat sample.

	HiBalance (*n* = 48)	Control (*n* = 47)
Age (years)^a^	71 (5.9)	71.1 (6.3)
Sex		
Female^b^	18 (37.5%)	17 (36.2%)
Male^b^	30 (62.5%)	30 (63.8%)
Body mass index^a^	25.3 (3.5)	25.4 (3.6)
Years of education^a^	15.1 (3.1)	14.3 (3)
Cohabiting^a^	38 (79.2%)	31 (66%)
Disease duration, years since diagnosis^c^	5.5 (7)	3 (4)
On dopaminergic therapy^b^	46 (95.8%)	46 (97.9%)
Levodopa equivalent dose (mg)^c^	551 (604.75)	450 (277)
Levodopa^b^	43 (89.6%)	41 (87.2%)
Dopamine agonists^b^	25 (52.1%)	21 (44.7%)
Catechol-O-methyltransferase inhibitors^b^	13 (27.1%)	5 (10.6%)
Monoamine Oxidase Type *B* inhibitors^b^	14 (29.2%)	12 (25.5%)
Movement Disorders Society – Unified Parkinson’s Disease Rating Scale-III score^a^	31.2 (11.9)	31.8 (10.3)
Movement Disorders Society – Unified Parkinson’s Disease Rating Scale-Total score^a^	51 (18.8)	50.4 (15.5)
Hoehn and Yahr 2^b^	39 (81.2%)	34 (72.3%)
Hoehn and Yahr 3^b^	9 (18.8%)	13 (27.7%)
Montreal Cognitive Assessment score^a^	26.1 (2.3)	25.4 (2.5)

^a^Mean (SD).

^b^*n* (%).

^c^Median (IQR).

### Expectations and blinding

At week 3 of the intervention, participants in the two groups expected a similar level of symptom improvement as an effect of the intervention (HiBalance: *m* = 53.3%, SD = 23, active control group: *m* = 55.1%, SD = 20). The assessor was blinded to group allocation for 44 of the participants at the Mini-Balance Evaluation Systems Test (Mini-BESTest) assessment after the intervention. At nine of the Mini-BESTest assessments, the assessors correctly guessed the participant’s group allocation but only after finishing the assessment. At six of the Mini-BESTest assessments, the assessor knew (*n* = 5) or correctly guessed the participant’s group allocation before the assessment started. For 15 participants, blinding scores of the Mini-BESTest were not reported. See Supplementary Tables 2 and 3 for detailed information on participants’ expectations and blinding scores, respectively.

### Adherence and adverse events

Participants in both groups attended a median of 85% (*n* = 17) of the group trainings (IQR HiBalance = 25, IQR active control group = 23). The median of executed home-exercise occasions was 90% (*n* = 9) in both groups (IQR HiBalance = 20, IQR active control group = 65). Twenty-three per cent of the participants who followed through their intervention (HiBalance programme: 18%, active control group: 29%) reported that they that performed 70% or less of the home training programme occasions. Thirty-eight per cent of the participants who followed through the HiBalance programme reported that they had not intensified their home exercises, in contrast to our instructions. Diversions from the HiBalance and the active control intervention’s core components were reported for each training occasion. There were no systematic deviations detected during the inspection of the training reports.

Eight adverse events were reported during the HiBalance training sessions, seven of which were non-injurious falls, and one was a calf-muscle strain. No adverse events were reported during the active control group sessions. Five individuals in the HiBalance group and six individuals in the active control group reported to have increased their daily levodopa equivalency dose during the study period and one individual in each group reported a decreased daily dose (no significant group difference, *p* = 0.39). See Supplementary Table 4 for details on medication changes.

### Effects of the HiBalance programme

**Fig. 2:**
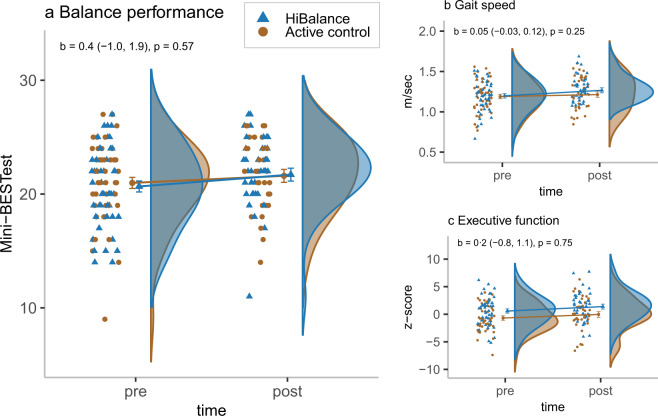
Effects of the HiBalance programme. The mean values, the standard error of the mean (error bars) as well as the *b* values, e.g., the unstandardised estimates of the time by group interaction, and their 95% CIs, are predicted values based on the intention to treat analyses. The participants’ point estimates, and their distributions are observed values. **a** Mini-BESTest (range 0–28), **b** Gait speed (m/s), **c** executive function (composite score of four tests from the Delis–Kaplan Executive Function System and the Wechsler Adult Intelligence Scale, *z*-scores).

There was no significant group by time interaction effect for our primary outcome, the Mini-BESTest (unstandardised *b* = 0.4 [95% CI = **−**1, 1.9], *p* = 0.57). The between-group Cohen’s *d* was estimated to be 0.14. There were also no significant group by time interaction effects in favour of the HiBalance programme for any of our secondary behavioural outcomes or the BDNF outcomes. There was however a significant group by time interaction effect on voice sound level (strength of the voice when reading a text out loud) where the active control group showed an increase in comparison to the HiBalance group (unstandardised *b* = −2.1 [95% CI = −3.4, −0.9], *p* = 0.0012) with the between-group Cohen’s *d* estimated to −0.54. See Fig. 2 and Table 2 for descriptive data and the analyses results of the behavioural outcomes and mBDNF. There were no significant group by time interaction effects in the fMRI data in either the striatum or within the large mask of frontal areas (the primary motor cortex, the premotor cortex, the supplementary motor cortex, the anterior cingulate cortex (ACC) and the dorsolateral prefrontal cortex (DLPFC)) or elsewhere in the brain (no significant clusters of voxels after correcting for the multiple statistical testing). As for the difference score correlations, there were no significant differences between the HiBalance group and the active control group, neither for the behavioural difference scores correlated with the fMRI data difference scores, nor for the behavioural difference scores correlated with the difference scores of the mBDNF values.

**Table 2:** Descriptive data (observed values) and analyses estimates (by intention to treat) for the behavioural outcomes and mBDNF.

	HiBalance group		Active control group		Time by group interaction			
	Pre	Post	Pre	Post	Reg.	*b* (95% CI)	*p*	Cohen’s *d*
Mini-BESTest^a^	20.7	22.1	21.0	21.8	lin.	0.4	0.57	0.14
	(3.4)	(3.0)	(3.5)	(3.1)		(−1.0, 1.9)		–
Gait speed (m/s)	1.2	1.3	1.2	1.2	lin.	0.05	0.25	0.25
	(0.2)	(0.2)	(0.2)	(0.2)		(−0.03, 0.12)		–
Step length (cm)	65.0	68.0	64.4	66.1	lin.	1.1	0.53	0.13
	(8.5)	(7.5)	(9.5)	(9.6)		(−2.3, 4.5)		–
Walk-12^b^	9.5	8.0	9.0	8.0	poiss.	−0.1	0.29	–
	(12.2)	(13.0)	(11.0)	(11.0)		(−0.4, 0.1)		–
Steps per day	4936.6	4800.8	5462.4	5609.5	log lin.	−0.1	0.47	–
	(3548.8)	(3631.0)	(4690.9)	(4580.9)		(−0.4, 0.2)		–
Frändin–Grimby^c^	3	3	3	3	binom.	0.9	0.30	–
	(1)	(1)	(1)	(1)		(−0.8, 2.6)		–
MDS-UPDRS-III^d^	31.2	29.1	31.8	28.8	lin.	−0.4	0.87	−0.038
	(11.9)	(11.9)	(10.3)	(10.7)		(−5.5, 4.6)		–
MDS-UPDRS Tot.^d^	51.0	48.2	50.4	45.8	lin.	−0.3	0.93	−0.020
	(18.8)	(17.8)	(15.5)	(16.8)		(−7.6, 6.9)		–
ABC^e^	84.2	87.2	84.1	88.1	beta	0.2	0.27	–
	(22.5)	(14.0)	(21.6)	(14.7)		(−0.1, 0.4)		–
Executive function^f^	0.6	1.5	−0.7	0.1	lin.	0.2	0.75	0.054
	(2.6)	(2.6)	(2.4)	(3.0)		(−0.8, 1.1)		–
mBDNF (pg/mL)	38,010.8	37,169.4	37,805.3	35,945.8	lin.	106.8	0.94	0.036
	(7956.7)	(5928.3)	(8044.6)	(6208.5)		(−2752.6, 2966.3)		–
Voice sound level (dB)	70.3	70.6	70.8	73.0	lin.	−2.1	0.0012	−0.54
	(3.5)	(3.9)	(4.0)	(4.0)		(−3.4, −0.9)		–
PDQ-39^g^	19.7	17.5	17.8	11.9	poiss.	−0.1	0.56	–
	(17.8)	(15.7)	(17.9)	(18.1)		(−0.4, 0.2)		–
EQ-5D VAS^h^	70	75	80	80	beta	0.2	0.26	–
	(20)	(14)	(15)	(19)		(−0.2, 0.5)		–
HADS depression^i^	3	2	3	2	poiss.	−0.1	0.79	–
	(5)	(2)	(3)	(4)		(−0.4, 0.3)		–
HADS anxiety^j^	4	4	3	3	poiss.	−0.1	0.38	–
	(5)	(4)	(3)	(6)		(−0.5, 0.2)		–

The pre and post values are mean and standard deviation for all normally distributed outcomes (reg. = lin.) and otherwise median and interquartile range. The column Reg. defines the type of multilevel model (mlm) used for the outcome. *lin.* linear, *log lin.* linear mlm on logged values, *binom.* logistic, *poiss.* Poisson, *beta* mlm based on beta regression. *b* unstandardised estimate. Degrees of freedom are not reported as the calculation is controversial and error prone for multilevel models. *Mini-BESTest* the Mini-Balance Evaluation Systems Test, *Frändin–Grimby* the Frändin–Grimby scale, *MDS-UPDRS* Movement Disorders Society – Unified Parkinson’s Disease Rating Scale, *ABC* the Activities-specific Balance Confidence Scale, *mBDNF* mature BDNF, *PDQ-39* the Parkinson’s Disease Questionnaire-39, *EQ-5D-VAS* the EuroQol-5 Dimensions visual analogue scale, *HADS* the Hospital Anxiety and Depression scale.

^a^Higher scores reflect better balance.

^b^Higher scores reflect more gait-related problems.

^c^Higher scores reflect a higher degree of physical activity in daily life.

^d^Higher scores reflect more Parkinson’s disease-related symptoms.

^e^Higher scores reflect higher balance confidence.

^f^Higher scores reflect higher executive function.

^g^Higher scores reflect a higher Parkinson’s disease-specific health-related quality.

^h^Higher scores reflect a better general health status.

^i^Higher scores reflect higher levels of depression.

^j^Higher scores reflect higher levels of anxiety.

**Fig. 3:**
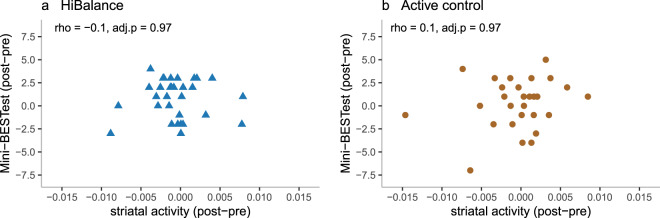
The difference score correlations of the Mini-BESTest and striatal activity. rho = Spearman’s rank-order correlation coefficient. adj. *p* = false discovery rate adjusted *p* (alpha = 0.05). **a** The difference score correlations in the HiBalance group. **b** The difference score correlations in the active control group.

The results of the per-protocol analyses, i.e., of participants who attended at least 60% of training occasions, also showed non-significant group by time interactions for all outcomes. See Fig. 3, Supplementary Tables 5 to 9 and Supplementary Fig. 1 for details on the difference score correlations, details of the intention-to-treat as well as the per-protocol analyses and *f*-value brain maps.

## Discussion

This double-blind, randomised controlled trial (RCT) involving individuals with mild to moderate PD, investigated the HiBalance programme in terms of behavioural outcomes and related changes in task-evoked brain activity and the neurotrophic factor BDNF. We used an active control group that induced similar levels of participant rated expectations and social interaction as the HiBalance programme, and successful blinding of the assessors. We did not find a significant group difference for our primary outcome of balance performance and the effect size was estimated to be rather small. Neither did we find evidence that the HiBalance programme resulted in significant group differences for our secondary measures including gait variables, executive function and relations between the changes in the behavioural outcomes and the changes in fMRI and BDNF.

Our results including the estimated effect sizes (e.g., Cohen’s *d* Mini-BESTest = 0.14) contrast a recent meta-analysis where balance and gait focused interventions for people with PD were concluded to have significant and rather large effects on motor symptoms and balance and gait difficulties (e.g., standardised mean difference post intervention Mini-BESTest = 0.90)^7^. The results also contrast with our earlier studies where the HiBalance programme was estimated to have significant positive effects on balance performance, gait speed, and dual-task ability with larger effect sizes than in the present study (e.g., Cohen’s *d* Mini-BESTest = 0.82 and 1, respectively^12,16^).

A possible explanation for the discrepancy in study results is sample characteristics. The sample in the present study had milder general motor symptoms than in our previous RCT and in our implementation study of the HiBalance programme and fewer balance impairments than in our previous RCT. This is likely the effect of the added MRI exclusion criteria and the extended measurement battery used in the present study. In this context, it is interesting to focus on the distribution of the gait speed estimates (Fig. 2, upper right corner) at the post-intervention assessments. In contrast to the pre-intervention assessment, there are no individuals in the HiBalance group who are at the bottom part of the scale, i.e., have a gait speed under 1.07 m/s, at the post-intervention assessment. There is no such pre to post assessment change in the distribution of gait speed estimates in the active control group. This is a possible indication that individuals with a lower gait speed benefit to a larger extent from the HiBalance programme. This hypothesis is also in line with a previous responsiveness study of the HiBalance programme that showed that participants who are more affected by their PD benefit to a greater extent from the programme^19^. The present study also differs from our previous RCT whereby group training occasions were reduced from three to two times per week to ease implementation in clinical settings. The third weekly session was substituted with a guided but unsupervised home-exercise session. Because 18% of the HiBalance participants reported to have performed less than 70% of the home-exercise occasions and a third to not have intensified their home exercises, it is possible that the home-exercise programme was an inadequate substitute of a clinical group session. The importance of training frequency was also observed in our previous responsiveness study where participants with a higher attendance rate to the HiBalance programme gained larger effects^19^.

The absence of support for exercise effects in people with PD in comparison to an active control group is however in line with findings of two recently conducted similar-sized RCTs which also used blinded assessors^20,21^. Jung et al.^21^ investigated an intervention with similar components and number of group exercise occasions (*n* = 18) as in the present study, in a somewhat similar sample (although assessed OFF medication). They did not find significant exercise effects on balance performance in general, motor ability or cognitive function. van der Kolk et al.^20^ investigated the effects of aerobic exercise over a 6-month period in a sample of people with milder PD. They found a significant exercise effect on physical fitness (VO_2_ max) and UPDRS-III in the OFF state of dopaminergic medication, driven by an increase in motor symptoms in the active control group. There was however no significant exercise effect on the UPDRS-III in the ON state of dopaminergic medication, or on any of their other secondary outcomes. The occurrence that several studies with blinded assessors and active control groups have not been able to find positive results, could indicate that earlier reported positive effects of physical exercise in PD have been inflated. This could be the effect of confounders such as assessment bias and non-specific factors of participating in an intervention such as participants’ expectations for symptom amelioration and social interaction.

Encouraged by earlier positive findings of physical exercise in people with PD^7–9,12^, we included measures of exercise-related changes in task-induced brain activity and BDNF in the present study. We hoped to provide insight into the mechanisms of symptom reduction by investigating associations between behavioural changes and brain-related changes. Because we did not find any significant or large effects of the HiBalance programme on our behavioural outcomes, the non-significant findings in both the fMRI data and the BDNF data were expected. We encourage future studies to further investigate brain-related changes of physical exercise in people with PD, when clinically relevant effects of a specific intervention have been repeatedly found in high-quality studies.

The quality of studies of physical exercise for people with PD has improved in recent years, including the use of larger samples, blinding of assessors and controlled designs^20,21^. The non-conclusive results of our studies of the HiBalance programme and other researchers’ studies of different types of physical exercise demand that the positive trend with well-designed studies continues. The strengths of the present study include that it was well-powered to detect a group difference of an interesting size, that we used assessor blinding and an active control group and that we also evaluated these quality characteristics. Our blinding was successful in that the group allocation was unknown for the assessor in most of the assessments. The design of our active control group was supported by the high expectations for symptom alleviation reported by both groups. The significant increase in voice sound level as an effect of the HiCommunication programme further strengthens the validity of using this intervention as the active control group. The positive effect on voice sound level is plausibly a result of a continuous focus on voice sound level in the HiCommunication intervention. These encouraging results will be investigated in further detail elsewhere.

There are some limitations of the present study. Twenty participants (21%) discontinued their allocated exercise programme, resulting in missing data. Multiple imputation and multilevel models were used to make the best use of the existing data. Unfortunately, these statistical methods were not feasible to use for the missing fMRI data where we lost additional data from 15 participants due to discomfort, low data quality etc. This meant that fMRI data was only available for a subsample of 60 participants, resulting in a decreased statistical power for these outcomes. Another limitation concerns the use of BDNF as a measure of neuroplasticity. BDNF is considered important for neural regeneration and neural protection. One should however be aware that measures of BDNF levels are only indirect measures of neuroplasticity. The changes in medications made by some participants deserve attention. Because the changes do not differ over the groups, they do not affect our between-group analyses but for future within-group analyses, changes in medication are a possible confounder. Lastly, and as already discussed, it was unfortunate that adherence to and progression of the home-exercise programme was not achieved by a relatively large proportion of participants. We believe that future implementation of e-health tools might facilitate the use of home-exercise programmes through increased motivation, reminders or possibilities for further instructions.

The research field of physical exercise for people with PD now spans a broad range of interventions with differences in the type of exercise, intensity, and length, investigated in samples of varying symptom severity. Meta-analyses have overall reported positive effects of physical exercise for people with PD, but the estimated effect sizes differ substantially over both outcomes and exercise type^7–9^. The great flora of exercise programmes developed for people with PD offers future studies an ample possibility to find the interventions most effective through high-quality systematic investigations. It is possible that a specific exercise intervention is the most effective intervention for most people with PD but because PD is a disease with great heterogeneity, it could also be the case that different types of interventions are needed for specific groups or even individuals with PD, depending on their symptom profiles.

In conclusion, we did not find support for positive effects of the HiBalance programme on the primary outcome of balance performance or on any of the secondary outcomes, in our sample of individuals with mild to moderate PD. The lack of statistically significant results and the size of the effects contrast with a recent meta-analysis of gait and balance focused exercise in people with PD and with the findings of our earlier studies of the HiBalance programme. The non-significant results are however congruent with two recent double-blind RCTs of a similar size as the present RCT. If future studies keep important quality aspects in focus, we believe that there is a substantial potential to further develop successful physical exercise interventions for people with PD. However, the divergent results raise important questions on how to optimally target and adjust training programmes to the included individuals and their symptoms, functional impairments, and needs. Other questions concern the optimal duration and frequency of the interventions. We propose that future studies investigate the effects of interventions specifically designed with regard to the symptom heterogeneity of PD, i.e., personalised rehabilitation programmes targeting specific symptom profiles or individuals. We also strongly encourage systematic investigations of the moderating effects of disease severity, intervention length and intensity, as well as exercise modality.

## Methods

### Study design

The EXPANd Trial was designed as a double-blind RCT (registered at clinicaltrials.gov NCT03213873), see also study protocol (https://pubmed.ncbi.nlm.nih.gov/31718583/)^22^. Assessors were blind to group allocation and participants were unaware that the HiCommunication programme served as an active control group. Both interventions were performed in a university hospital setting. The feasibility of the study design was evaluated in a pilot study^23^. The trial was approved by the Regional Ethical Review Board in Stockholm 2016/1264–31/4, 2017/1258–32 and 2017/2445–32.

### Participants

Participants were recruited in four successive waves from 2018 to 2019 via advertisements in local newspapers and through the Swedish Parkinson Association. Following an initial telephone screening, eligibility was established at an in-person assessment in a university setting. Participants with mild to moderate idiopathic PD were eligible for inclusion if they were ≥60 years of age, at Hoehn and Yahr stage 2 or 3^24^, scored ≥21 on Montreal Cognitive Assessment^25^, had been stable in anti-Parkinson’s medication for approximately 3 weeks, were able to ambulate indoors without a mobility aid, and scored ≤27 on the Mini-BESTest^26^. Participants were excluded if they had any other disorder that substantially influenced balance, voice or speech performance, had taken part in an exercise programme for balance or speech during the last 6 months, had MRI incompatible implants, claustrophobia, uni- or bilateral blindness, an inability to hear instructions without a hearing aid, or suffered from severe states of tremor, dyskinesia, dystonia or diplopia. The assessment of disease severity was conducted by physiotherapists experienced in people with PD and symptom assessment by Hoehn and Yahr. All participants provided written informed consent.

### Randomisation and masking

For each consecutive wave, participants who met all eligibility criteria were randomly allocated (1:1) to the HiBalance programme or the active control group. The randomisation was based on a true random number service (http://www.random.org) and performed by an individual not responsible for assessment or data analysis. The participants were informed of their group allocation through sealed opaque envelopes. All assessors were blinded to group allocation, and participants were instructed not to disclose any information of their programme content during the post-intervention assessments. The assessors reported their perceived level of blinding after each assessment by use of a questionnaire. The blinding was kept throughout the statistical analyses using arbitrary group indicators. Participants were informed that the purpose of the study was to compare the effects of the HiBalance programme and the HiCommunication programme, but not that the latter was considered a control intervention. The participants rated their expectations and the credibility of the allocated intervention three weeks into the intervention.

### Procedures

The data collected during the first in-person assessment included baseline characteristics such as sex and age, height, weight, levodopa medication, information on prior falls, walking aids and other diseases/disorders, assessments of balance performance, gait and motor function and self-reported data on walking ability, physical activity, balance confidence, health-related quality of life and symptoms of depression and anxiety. The data collection for included participants continued with one session of brain imaging with MRI, one session with assessments of cognitive functions and speech and voice function and blood sampling. All assessment sessions were performed during ON state and scheduled on separate days to avoid fatigue. Each person’s post-intervention assessments were scheduled at approximately the same time of the day as the baseline assessments. Participants were advised against making changes in medication or to their regular physical exercise routines during the study period.

The HiBalance programme is a group training intervention where participants perform highly challenging balance exercises intended to improve four core areas of balance control: (I) sensory integration, (II) motor agility, (III) anticipatory postural adjustments and (IV) stability limits. The HiBalance methodology incorporates principles of motor learning (i.e., specificity, progressive overload and variation) and trainers adapt each task individually in order to ensure that the exercises are highly challenging. The difficulty level is further increased by the introduction of both cognitive (e.g., counting backwards) and motor dual tasks (e.g., carrying a tray with balls). Two physiotherapists are present at all group training sessions to minimise fall risk. More details on the core components and the progressive tasks in the HiBalance programme can be found in Table 3 and elsewhere^22,23^.

**Table 3:** Description of the core components and the progression of the HiBalance programme and the active control group programme.

	HiBalance programme	HiCommunication programme Control group
*Core components*	Sensory integration Anticipatory postural adjustments Motor agility Stability limits	Voice sound level Articulatory precision Word retrieval Memory
*Progression*		
Block A Weeks 1–2	Exercises were performed with a focus on movement quality, familiarisation of the exercises and task-specific motor learning. Single task performance of exercises pertaining to each of the core components	Exercises were performed with a focus on phonation, articulation and breathing. Increased vocal loudness was established while maintaining good voice quality
Block B Weeks 3–6	Increased level of difficulty and complexity of the exercises was established through variation of the exercises within the core components and by introducing cognitive and motor dual tasks	Increased level of difficulty and cognitive load during the exercises was established by the introduction of memory games and associational tasks
Block C Weeks 7–10	Complexity further increased through task variation, by combining exercises from all four core components, and by integrating simultaneous cognitive and motor dual tasks	Complexity further increased by the enhanced difficulty of memory games, by incorporating more interaction between participants and by adding background noise

The HiCommunication programme was led by speech-language pathologists and the exercises performed were aimed to improve four core areas of relevance for speech and communication: (I) voice sound level, (II) articulatory precision, (III) word retrieval and (IV) memory. As in the HiBalance programme, the intervention was performed in groups, but with individual adaptation by the trainers to ensure that the exercises were highly challenging. The difficulty level was further increased through the addition of background noise, challenging memory tasks and tasks requiring communicative interaction between participants. The intervention was based on principles suggested to promote experience-dependent neuroplasticity^23,27^. More details on the core components and the progressive tasks in the active control group can be found in Table 3.

Both interventions were delivered with the same dose (10 weeks), frequency (twice per week), length (60 min/session) and group size (6–8 participants). The active control group performed all speech- and communication exercises in a seated position. Both training interventions also included a home-exercise programme to be performed once a week. These home-exercise programmes had a focus on functional aerobic and strength exercises in the HiBalance group, and a focus on voice and speech function in the active control group.

Attendance and adverse events were monitored during the group training sessions, and compliance to the home-exercise programme was overseen using diaries. The trainers’ fidelity to the HiBalance programme core components was monitored through inspection of reports filled out by the trainers after each session.

### Behavioural outcomes

The primary outcome was balance performance assessed with the Mini-BESTest, a rating scale for dynamic balance validated in people with PD^26,28^. The 14-item clinical test covers four components of balance control and has a maximum score of 28 points with higher scores indicating better balance control. Three gait-related variables that together illustrate key aspects of gait deficits in people with PD were included as secondary outcomes: comfortable gait speed and step length assessed on an electronic walkway (GAITRite^®^, CIR Systems, Inc., Havertown, PA, USA), and self-reported gait ability (the Walk-12 scale^29^). Habitual physical activity (steps per day) was measured by an accelerometer (Actigraph GT3X+, Pensacola, FL, USA) for 7 consecutive days, and self-reported level of physical activity through the Frändin–Grimby scale^30^. Various motor and non-motor aspects of PD were captured using the total score on the Movement Disorder Society – Unified Parkinson’s Disease Rating Scale^31^, whereas motor function specifically was addressed through part III of the same scale. Balance confidence was reported using the Activities-specific Balance Confidence scale (ABC scale^32^). Executive function was assessed with a composite measure of three tests from the Delis–Kaplan Executive Function System^33^ (letter fluency and category switching from Verbal Fluency, and the switch condition from the Color-Word Interference Test), and one test measure from the Wechsler Adult Intelligence Scale^34^ (Digit Span total score). Recordings of speech and voice were used to investigate the effects of the HiCommunication training. The recordings were performed according to standardised routines for high-quality recordings in a sound-proof recording studio with the equipment Sony Digital Audio Tape Deck DTC-ZE700 and the software Sopran (version 1.0.22 © Tolvan Data). The outcome measure from the studio recordings used in the present study was mean voice sound level (dB SPL) in reading a Swedish standardised text. Self-reported data on health-related quality of life was collected using Parkinson’s Disease Questionnaire-39^35^ and EuroQol-5 Dimensions-VAS (EQ-5D^36^) and symptoms of depression and anxiety were measured with the Hospital Anxiety and Depression scale^37^.

Attendance and adverse events were monitored during the group training sessions, and compliance to the home-exercise programme was overseen using diaries. The trainers’ fidelity to the HiBalance programme core components was monitored through inspection of reports filled out by the trainers after each session.

### fMRI outcomes

Indirect measures of brain activity were acquired by fMRI and the blood-oxygen-level-dependent signal. A 3T Phillips Ingenia scanner with a 15-channel head coil with the following parameters was used: repetition/echo time = 2085/35 ms, flip angle = 75°, voxel-size: 3.5 × 3.5 × 3.5 mm, field of view: 224 × 224 × 140, 265 slices in ascending order.

The fMRI data were acquired during a computer-based motor learning task named the serial reaction time task developed by Nissen and Bullemer^38^ and modified in a feasibility study by our research group^39^. The serial reaction time task has the benefit of requiring actual motor action but only minimal finger movements, unlikely to induce head movements to a great extent. Furthermore, the task measures the important ability of implicit motor learning. Implicit motor learning is frequently reported to be impaired in people with PD and the impairment plausibly contributes to the gait and balance deficits^4,40^. The serial reaction time task measures implicit motor learning ability by assessing whether the participants learn a hidden sequence of visually presented stimuli, without awareness of learning it. The serial reaction time task used was 9 min long and presented in Psychopy (version 1.85.4). Four white circles on a horizontal line were shown on a black screen and each circle’s position corresponded to one out of four buttons of two response pads (two buttons per response pad). Every 1.2 s, one of the circles turned grey and the participant was to press the corresponding button as quickly as possible. The serial reaction time task consisted of ten blocks of trials; each block interleaved by a 6-s break. Unbeknownst to the participants, in six of the ten blocks, the trials followed a 10-item higher-order sequence. Two different sequences but with the same characteristics were used for the pre and post assessment, respectively. Before performing the serial reaction time task in the scanner, the participants practised the serial reaction time task seated at a table outside the scanner room, using the same type of response pads as used in the scanner. The training version of the serial reaction time task consisted solely of random trials. The training ended when the participant achieved 80% accuracy (after at least two rounds of training) or after a maximum of five rounds. All files needed to run the task can be found at https://osf.io/s952g/.

Initial quality control (QC) of MRI data was done using MRIQC and the pre-processing was done using fMRIPrep^41,42^. When available, the two T1 images from each individual’s pre and post scans were merged and used as a longitudinal template for coregistration with the functional images. Mapping to standard space was done using the MNI template 2009c. For individuals with field maps, these were included in the fMRIPrep pipeline. A boilerplate for the pre-processing made with fMRIPrep can be found here: https://osf.io/s952g/. Smoothing was used using SPM12 default process with smoothing at 8 mm FWHM. Authors M.F. and W.H.T. assessed the coregistration of the fMRI images to the T1 images and an initial check of signal drop-out using the output of MRIQC. No participant was excluded due to low-quality coregistration. Two participants lacked more than 80% of the voxels in the striatum (as defined by our atlas of the striatum) due to signal drop-out and were excluded from the analyses of striatal activity. Thirty-five participants had missing fMRI data or too low-quality fMRI data. The reasons were drop out (*n* = 20), possibly incompatible implants/metal splitter (*n* = 2), head did not fit in coil/no room for the mirror showing the task (*n* = 3), pain/discomfort (*n* = 4), technical problems (*n* = 3) and framewise displacement >0.5 (*n* = 3).

Group-level effects of the HiBalance programme on fMRI data were investigated separately within the striatum and within one large region of interest including the primary motor cortex, the premotor cortex, the supplementary motor cortex, the ACC, and the DLPFC. These brain regions were chosen based on their involvement in PD processes, motor, and cognitive functions^2,43,44^. In addition, the changes in fMRI between the pre- and post-assessment were correlated with the change between the pre- and post-assessments for the outcomes of balance, gait speed and executive function. The automated anatomical atlas 3^45^ was used to create masks for the extraction of data, the striatum and ACC. The Human Motor Area Template atlas^46^ was used to create masks for the primary motor cortex, the premotor cortex and the supplementary motor cortex. Brodmann area 46 was used to create a mask to extract data from the DLPFC.

### BDNF outcomes

Blood samples were collected to analyse serum levels of BDNF. The blood samples were obtained in a hospital setting, centrifuged, aliquoted into 1 mL tubes, and stored at −80 °C to retain protein integrity. In advance of the testing day, the serum was thawed slowly on wet ice, aliquoted into 5 × 200 µL Eppendorf tubes; placed on dry ice, and then refrozen to −80 °C within 15 min of thawing. Thaw and refreezing cycles, when aliquoting and preparing samples for testing, were consistent for all samples. On the day of testing (analyses of the samples), the samples were thawed at room temperature, diluted with assay diluent, and loaded into the ELISA plates via multichannel pipettes within 1 h of thawing. Two separate ELISA kits were used for the quantification of proBDNF and mBDNF. Both ELISA kits used a sandwich format. A recombinant BDNF protein standard curve was used to extrapolate the values of the unknown samples (concentrations unknown). This allowed for samples with low concentrations to be interpolated by GraphPad Prism 8.4.2, as proBDNF is often low quantity or undetectable. Samples, along with the standards and spike (used as an accuracy measure) were loaded into 96 clear-welled and flat-bottomed microplates pre-coated with capture antibodies. The unknown samples were randomised and arranged on ELISA plates so that every assay included a selection of paired samples (pre- and post-intervention serum samples) and unpaired samples (either pre- or post-intervention serum samples). The standard curve, blank and positive control (PC)/QC were consistently positioned on each plate. Microplates were then read to acquire sample absorbance values with a TECAN Spark^®^ 10M multimodal microplate reader.

A commercially available proBDNF ELISA Kit (Rapid™ ELISA), compatible with human proBDNF, was sourced from Biosensis^®^ (Catalogue Number: BEK-2237-1P/2P). The standard rage fell between 15.6 and 1000 pg/mL. Maximal sensitivity range was not specified in the protocol. Three standard curve points (7.8, 3.9 and 2.0 pg/mL) were added, providing the lowest standard curve concentration. The proBDNF was a recombinant product produced and validated by Biosensis^®^. Cross-sensitivity with human mBDNF was stated at <0.3% (w/v). A recombinant, 350–650 pg/mL, proBDNF QC (PC) sample served as an inter/intra-plate performance evaluator. Capture antibody paratope was specific to pro-domain epitope, thus targeting full-length BDNF (proBDNF) and avoiding cross-detection with mBDNF.

A commercially available mBDNF ELISA kit (Aviscera Bioscience; BDNF (Human, Mouse, Rat) Catalogue Number: SK00752-01) compatible with human BDNF was used. The standard range, as stated by the protocol, fell between 23 and 1500 pg/mL, with a maximal sensitivity between 5 and 8 pg/mL. Kits were compatible with both human serum and plasma. Cross-sensitivity with human proBDNF was stated at <1% (w/v). Capture antibody paratope was specific to a mature-domain epitope, thus targeting short-length BDNF (mBDNF) and avoiding cross-detection with proBDNF.

### Statistical analyses

An independent statistician performed a power calculation using 2000 bootstrap samples and the variance estimates from our pilot study^23^. By testing a random-intercept model with group, time and their interaction as covariates and the alpha level set to 0.05 (two-sided), it was estimated that a sample size of 40 individuals per group would result in a power of 82% to detect a between-group difference of two points in the mean of the total score of the Mini-BESTest at post assessment. The two-point difference was based on the effect of similar intervention studies^12,16^ and the measurement error of the Mini-BESTest^28^. To account for drop-outs and data exclusion due to technical problems or low imaging quality, we aimed for 50 participants in each group.

The composite score of executive function was based on four tests: letter fluency, the verbal fluency test: category switching, the colour-word interference test: switch condition (all three tests from the Delis–Kaplan Executive Function System) and the digit span total score from Wechsler Adult Intelligence Scale and was created in several steps. First, the pre and the post-assessment scores of each of the four tests were standardised into *z*-scores using the mean and standard deviation of the scores obtained pre the intervention. Second, four models were compared using the standardised pre-assessment values: with and without including the colour-word interference test: switch condition and using either the maximum likelihood estimation or the robust diagonally weighted least square estimation. Models were tested with and without the colour-word interference test: switch condition because it had a skewed distribution. The model including all four tests and using the robust diagonally weighted least square estimation was chosen based on fit values (robust RMSEA = 0.044, robust Comparative Fit Index = 0.995, robust TLI = 0.984). Factor loadings of the model: verbal fluency = 1.000, the verbal fluency test: category switching = 0.887, the colour-word interference test: switch condition = −0.855, the digit span total score = 0.813. Last, the *z*-scores of each test and person were multiplied with the factor loadings and the resulting test scores for each test were added together to create a sum score measure for each person and assessment time point, respectively.

Our primary analyses of the behavioural outcomes and mBDNF included data from all participants with missing values imputed using multiple imputation. We used the R package mice and the predictive mean matching method with 30 imputed data sets, 10 iterations and data separately imputed for the two groups. Predictors for each outcome were chosen based on theoretical assumptions in combination with correlation coefficients (Supplementary Table 1). Diagnostic plots were used for the evaluation of the imputations.

Linear or generalised multilevel models were used depending on the distributions of the residuals and model fit. Group (HiBalance = 1, active control = 0) and time (pre = 0, post = 1) and their interaction were used as predictors. The alpha level was set to 0.05, two-sided. We performed complementary per-protocol analyses of the behavioural outcomes and mBDNF, where solely participants who attended at least 60% of the training occasions were included. For both the intention to treat analyses and the per-protocol analyses, the models were specified with the pre and post values as level 1, clustered within the individuals, i.e., level 2, with group as a factor on level 2. We allowed for random intercepts but not random slopes (due to unstable values when there are only two time points). Time and group and their interaction were used as independent variables. No covariates were included. The restricted maximum likelihood estimation was used. As there is no consensus on how to correctly calculate the degrees of freedom for multilevel models and suggested methods are error prone, we refrain from reporting the degrees of freedom. For the intention to treat analyses (except the ABC and EQ-5D scale), the multilevel models were estimated on each imputed data set, followed by pooling of the estimates as implemented by the R package mice. The ABC scale and the EQ-5D were left-skewed and therefore multilevel models were used with a specification of a beta-distribution using the R package glmmTMB. As there is no tool available to pool the results of multilevel models with a defined beta-distribution, the multilevel models on the ABC scale and the EQ-5D were done on non-imputed data but still using the intention to treat approach, i.e., *n* = 95. A multilevel model was also used to analyse the data of the serial reaction time task but with a different specification due to the data structure. The predictors were trial number, type of block (sequence/random), time (pre/post), group, all two-way and three-way interactions of the type of block, time, and group. Type of sequence and time were clustered within participant (level 2), with group as a factor on level 2. We specified random intercepts and random slopes over the trial numbers. The restricted maximum likelihood estimation was used.

The standardised effect size Cohen’s *d* was calculated for the normally distributed behavioural outcomes and mBDNF. We used the *b* coefficient of the time group interaction as the nominator and the pooled SD ((SD HiBalance + SD Active Control)/2) of the observed values post the intervention, as the denominator. Cohen’s *d* is not suitable for non-normal distributions and was therefore only calculated and reported for the outcomes that were approximately normally distributed.

For the first-level analyses of the fMRI data, the independent variables were the experimental timeline convoluted with the canonical hemodynamic function, 24 motion-derived regressors as well as the first five aCompCor regressors and the cosine regressors. Activity during random blocks was contrasted to activity during sequence blocks, creating statistical contrast maps for each individual and scan. Group-level analyses were performed using the flexible factorial model as implemented in SPM12^47^. The group-level analyses were performed separately for the striatum and for one mask comprising of multiple regions of interest that included the primary motor cortex, the premotor cortex, the supplementary motor cortex, the ACC, and the DLPFC. A cluster-defining threshold of *p* = 0.05, family-wise error corrected, was used.

Spearman’s rank-order correlation was used to estimate the correlations between the difference scores (pre/post assessment) of balance ability, gait speed and executive function with the difference scores of the fMRI data (mean of the top 10% most active voxels in each of the selected brain areas when for values larger in the sequence blocks than in the random blocks^48^) and mBDNF, within the HiBalance and the active control group, respectively. We then used Fischer’s significance test by first transforming the correlations coefficients to *z*-scores and then significance testing the transformed correlations coefficients over the two groups. False discover correction was done separately for our more primary hypotheses, i.e., the correlations between mature BDNF and striatal activity with the behavioural outcomes, respectively, and the remaining correlations.

We used R 4.0.3. for the multiple imputation, the statistical group analyses of the behavioural outcomes and BDNF outcomes as well as for the difference score correlations. We used SPM12 (version 7771) for the first- and second-level analyses of the fMRI data. Our prespecified plan for the statistical analyses as well as scripts of the statistical analyses can be found at https://osf.io/s952g/.

### Deviations from the analysis plan

The original plan (https://osf.io/s952g/) was to use multilevel models also for the brain activity data and thereby enable the inclusion of participants for whom we lost either the pre or post fMRI data. Due to technical difficulties, these multilevel analyses were not feasible to perform on the data and instead we used the conventional flexible factorial model as implemented in SPM12. We also used the default SPM12 smoothing method rather than structure adaptive smoothing.

### Reporting summary

Further information on research design is available in the Nature Research Reporting Summary linked to this article.

## Supplementary information

Supplementary Information

Reporting Summary

## Data Availability

With respect to the Swedish and EU personal data legislation (GDPR), the data are not freely accessible due to regulations regarding personal integrity in research, public access and privacy. The data are available from the principal investigator of the project: Erika Franzén (erika.franzen@ki.se), on a reasonable request. Any sharing of data will be regulated via a data transfer and user agreement with the recipient.

## Code availability

All scripts of the statistical analyses performed can be found at https://osf.io/s952g/.
