# Supplementary material for: Behavioural and neuroplastic effects of a double-blind randomised controlled balance exercise trial in people with Parkinson’s disease
Source: NPJ Parkinsons Dis. 2022 Jan 21;8:12. doi: 10.1038/s41531-021-00269-5 (PMC8782921; doi:10.1038/s41531-021-00269-5)
Supplement: Supplementary file 1 — Supplementary Information [file 41531_2021_269_MOESM1_ESM.pdf]

Supplementary material to “Behavioural and neuroplastic effects of a double-blinded randomised controlled exercise trial in people with Parkinson’s disease”

Table of Contents

Supplementary Table 1. Correlation matrix of all behavioural and BDNF outcomes. 2

Supplementary Table 2. Credibility assessments and expectations of the two interventions. 3

Supplementary Table 3. Blinding of the assessors at the post intervention assessments. 4

Supplementary Table 4. Medication changes over the course of the intervention. 4

Supplementary Table 5. Estimates of the MLM analyses based on the imputed data (by intention to treat). 5

Supplementary Figure 1: The f-values map of the striatum from the time by group interaction analysis. 6

Supplementary Table 6. Difference score correlations within the groups between the behavioural outcomes and the fMRI data and mBDNF. 7

Supplementary Table 7. Comparison of the difference score correlations between the behavioural outcomes and brain activity and mBDNF, over the groups. 7

Supplementary Table 8. Estimates of the MLM analysis of the reaction time outcome of the serial reaction time task (motor learning task performed during fMRI). 8

Supplementary Table 9. Estimates of the MLM per protocol analyses. 9

Supplementary References 10

Supplementary Table 1. Correlation matrix of all behavioural and BDNF outcomes.

	1	2	3	4	5	6	7	8	9	10	11	12	13	14	15	16	17	18
**1.Mini-BESTest**	1	0.47	0.52	-0.56	0.38	0.26	-0.37	-0.42	0.53	0.26	0.35	-0.17	-0.07	0.03	-0.4	0.45	-0.29	-0.18
**2.Gait speed**	0.47	1	0.89	-0.49	0.45	0.25	-0.29	-0.34	0.44	0.26	0.14	-0.08	-0.12	0.11	-0.22	0.37	-0.32	0.06
**3.Step length**	0.52	0.89	1	-0.5	0.49	0.25	-0.38	-0.39	0.47	0.22	0.14	-0.12	-0.12	0.12	-0.27	0.43	-0.34	-0.01
**4.Walk-12**	-0.56	-0.49	-0.5	1	-0.34	-0.25	0.39	0.63	-0.79	-0.21	-0.25	0.03	0.09	0.01	0.72	-0.73	0.69	0.48
**5.Steps/day**	0.38	0.45	0.49	-0.34	1	0.25	-0.23	-0.33	0.27	0	0.08	-0.13	-0.09	0.03	-0.24	0.23	-0.2	-0.04
**6.Frändin-Grimby**	0.26	0.25	0.25	-0.25	0.25	1	-0.11	-0.16	0.07	0.13	0.22	-0.11	0	-0.17	-0.11	0.25	-0.23	-0.05
**7.MDS-UPDRS III**	-0.37	-0.29	-0.38	0.39	-0.23	-0.11	1	0.82	-0.26	-0.21	-0.31	0.22	0.13	-0.23	0.2	-0.23	0.21	0.02
**8.MDS-UPDRS total**	-0.42	-0.34	-0.39	0.63	-0.33	-0.16	0.82	1	-0.51	-0.15	-0.24	0.2	0.22	-0.03	0.57	-0.5	0.54	0.38
**9.ABC**	0.53	0.44	0.47	-0.79	0.27	0.07	-0.26	-0.51	1	0.1	0.19	0.08	-0.16	-0.08	-0.65	0.63	-0.6	-0.45
**10.MoCA**	0.26	0.26	0.22	-0.21	0	0.13	-0.21	-0.15	0.1	1	0.56	-0.12	0.11	0	-0.07	0.08	-0.1	0.1
**11.Executive function**	0.35	0.14	0.14	-0.25	0.08	0.22	-0.31	-0.24	0.19	0.56	1	0.01	0.09	0.11	-0.14	0.13	-0.24	-0.1
**12.proBDNF**	-0.17	-0.08	-0.12	0.03	-0.13	-0.11	0.22	0.2	0.08	-0.12	0.01	1	-0.16	-0.04	0.06	-0.15	0.28	0.08
**13.mBDNF**	-0.07	-0.12	-0.12	0.09	-0.09	0	0.13	0.22	-0.16	0.11	0.09	-0.16	1	0.06	0.13	0.03	0.02	-0.04
**14.Voice sound level**	0.03	0.11	0.12	0.01	0.03	-0.17	-0.23	-0.03	-0.08	0	0.11	-0.04	0.06	1	0.04	0.01	0.11	0.01
**15.PDQ-39**	-0.4	-0.22	-0.27	0.72	-0.24	-0.11	0.2	0.57	-0.65	-0.07	-0.14	0.06	0.13	0.04	1	-0.65	0.66	0.74
**16.EQ5D, VAS**	0.45	0.37	0.43	-0.73	0.23	0.25	-0.23	-0.5	0.63	0.08	0.13	-0.15	0.03	0.01	-0.65	1	-0.65	-0.6
**17.HADS depression**	-0.29	-0.32	-0.34	0.69	-0.2	-0.23	0.21	0.54	-0.6	-0.1	-0.24	0.28	0.02	0.11	0.66	-0.65	1	0.65
**18.HADS anxiety**	-0.18	0.06	-0.01	0.48	-0.04	-0.05	0.02	0.38	-0.45	0.1	-0.1	0.08	-0.04	0.01	0.74	-0.6	0.65	1

All values are Pearson's correlation coefficients. The figures in the upper row correspond to the outcome name with the same figure presented in the left most column. Mini-BESTest = the Mini-Balance Evaluation Systems Test, Frändin-Grimby = the Frändin-Grimby scale, MDS-UPDRS = Movement Disorders Society - Unified Parkinson's Disease Rating Scale, MoCA = the Montreal Cognitive Assessment score ABC = the Activities-specific Balance Confidence Scale, proBDNF = precursor BDNF, mBDNF = mature BDNF, PDQ-39 = the Parkinson's Disease Questionnaire-39, EQ5D- VAS = the EuroQol 5 dimensions visual analogue scale, HADS = the Hospital Anxiety and Depression scale.

Supplementary Table 2. Credibility assessments and expectations of the two interventions.

	HiBalance m (sd)	Active control m (sd)	t-value	p-value	adjusted p-value
**How successful symptom alleviation**	6.6 (1.4)	5.8 (1.1)	-3.1	0.0026	0.0070
**Recommend to a friend**	7.6 (1.2)	6.5 (1.3)	-3.9	0.00021	0.0021
**How much better (%)**	53.3 (23)	55.1 (20)	0.4	0.69	0.69
**Challenging tasks**	5.5 (1)	5 (1.6)	-1.6	0.11	0.14
**Feels valuable to me**	7.5 (1.2)	6.6 (1.6)	-3	0.0035	0.0070
**Enhance my memory**	5.3 (1.9)	5.9 (1.4)	1.7	0.094	0.14
**Get to know my group members**	5.9 (1.6)	6.3 (1.3)	1.2	0.22	0.25
**Enhance my dual task ability**	6 (1.4)	5.3 (1.7)	-2	0.046	0.077
**Benefit**	7.5 (1.2)	6.7 (1.3)	-3.1	0.0027	0.0070
**Motivation**	8.1 (1)	7.4 (1.2)	-3	0.0035	0.0070

Questionnaire answered at week 3 to 4 into the interventions. Range 1-9 for all questions but “How much better” where percentage was used. The questionnaire was inspired by Borkovec and Nau. [15] P-values were adjusted using the false discovery rate with alpha = 0.05.

Supplementary Table 3. Blinding of the assessors at the post intervention assessments.

	Knew before n (%)	Guessed before n (%)	Correct guess before n (%)	No guess n (%)	Guessed after n (%)	Not reported n (%)
**Mini-BESTest**	5 (6.8)	1 (1.4)	1 (1.4)	44 (59.5)	9 (12.2)	15 (20·3)
**MDS-UPDRS**	3 (4·1)	0 (0)	0 (0)	49 (66·2)	2 (2·7)	15 (20·3)
**MRI**	3 (4·6)	4 (6·2)	1 (1·5)	24 (36·9)	32 (49·2)	12 (18·5)
**Speech, cognition**	6 (9·2)	9 (13·8)	8 (12·3)	43 (66·2)	9 (13·8)	6 (9·2)

All blinding scores were reported at the end of the assessment. Knew before = the assessor knew group allocation before the post assessment started, Guessed before = the assessor guessed group allocation based in information obtained before the post assessment started, Correct guess before = the assessor was right in the guessed group allocation based on information obtained before the post assessment started, No guess = the assessor had no guess on group allocation either before the start or at the end of the post assessment, Guessed after = the assessor obtained a guess on group allocation at the end of the post assessment, Not reported = missing blinding score. Mini-BESTest = the Mini-Balance Evaluation Systems Test, MDS-UPDRS = Movement Disorders Society - Unified Parkinson's Disease Rating Scale, MRI = magnetic resonance imaging.

Supplementary Table 4. Medication changes over the course of the intervention.

	HiBalance group (n = 40)	Active control group (n = 35)	t-value	p-value
**Number of participants who increased their medication during the intervention**	5	6		
**Increase mg, m (sd)**	89.6 (23.3)	105.7 (35.4)	0.4	0.39
**Number of participants who decreased their medication during the intervention**	1	1		
**Decrease mg (m)**	-93.0	-49.5		

mg = milligram, m = mean, sd = standard deviation.

Supplementary Table 5. Estimates of the MLM analyses based on the imputed data (by intention to treat).

		Group		Time		Group x Time		Intercept	
	**reg**	**b (95% CI)**	**p**	**b (95% CI)**	**p**	**b (95% CI)**	**p**	**b (95% CI)**	**p**
**Mini-BESTest**	lin.	-0.3 (-1.7, 1)	0.65	0.6 (-0.4, 1.6)	0.22	0.4 (-1, 1.9)	0.57	21 (20, 21.9)	< 0.0001
**Gait speed**	lin.	0.9 (-6.8, 8.5)	0.82	2.4 (-3.3, 8.1)	0.41	4.4 (-3.2, 12.1)	0.25	118.9 (113.5, 124.4)	< 0.0001
**Step length**	lin.	0.5 (-3, 4.1)	0.77	1.1 (-1.8, 4)	0.47	1.1 (-2.3, 4.5)	0.53	64.4 (61.9, 67)	< 0.0001
**Walk 12**	poiss.	0.2 (-0.2, 0.5)	0.31	0 (-0.2, 0.2)	0.89	-0.1 (-0.4, 0.1)	0.29	2.1 (1.9, 2.4)	< 0.0001
**Steps/day**	log lin.	-0.1 (-0.3, 0.2)	0.66	0 (-0.1, 0.2)	0.65	-0.1 (-0.4, 0.2)	0.47	8.5 (8.3, 8.7)	< 0.0001
**Frändin -Grimby**	binom.	-0.1 (-1.2, 1.1)	0.92	-0.5 (-1.6, 0.7)	0.43	0.9 (-0.8, 2.6)	0.30	-0.6 (-1.4, 0.3)	0.19
**MDS-UPDRS III**	lin.	-0.5 (-5.1, 4)	0.82	-1.9 (-5.5, 1.7)	0.30	-0.4 (-5.5, 4.6)	0.87	31.8 (28.5, 35)	< 0.0001
**MDS-UPDRS Tot.**	lin.	0.5 (-6.4, 7.5)	0.88	-3 (-8, 1.9)	0.28	-0.3 (-7.6, 6.9)	0.93	50.4 (45.5, 55.4)	< 0.0001
**ABC**	beta	-0.1 (-0.6, 0.4)	0.73	0.1 (-0.1, 0.3)	0.019	0.2 (-0.1, 0.4)	0.27	1.7 (1.3, 2.1)	< 0.0001
**Executive function**	lin.	1.3 (0.2, 2.4)	0.024	0.6 (-0.1, 1.4)	0.10	0.2 (-0.8, 1.1)	0.75	-0.7 (-1.5, 0.1)	0.090
**mBDNF**	lin.	196.3 (-3095.1, 3487.8)	0.91	-369.5 (-2409.5, 1670.6)	0.72	106.8 (-2752.6, 2966.3)	0.94	38071.9 (35714.7, 40429.1)	< 0.0001
**Voice sound level**	lin.	-0.5 (-2.1, 1.2)	0.58	2 (1.1, 2.9)	<0.0001	-2.1 (-3.4, -0.9)	0.0012	70.9 (69.7, 72)	< 0.0001
**PDQ-39**	poiss.	0.2 (-0.1, 0.6)	0.16	-0.2 (-0.4, 0.1)	0.16	-0.1 (-0.4, 0.2)	0.56	2.7 (2.4, 2.9)	< 0.0001
**EQ5D VAS**	beta	-0.4 (-0.9, 0)	0.044	0 (-0.2, 0.3)	0.037	0.2 (-0.1, 0.5)	0.26	1.3 (1, 1.7)	< 0.0001
**HADS Depression**	poiss.	0.1 (-0.3, 0.5)	0.59	0 (-0.3, 0.3)	0.95	-0.1 (-0.4, 0.3)	0.79	0.9 (0.7, 1.2)	< 0.0001
**HADS Anxiety**	poiss.	0.2 (-0.2, 0.5)	0.34	0.1 (-0.2, 0.4)	0.46	-0.1 (-0.5, 0.2)	0.38	1.1 (0.9, 1.4)	< 0.0001
**proBDNF (pg/ml)**	lin.	0.6 (-0.4, 1.6)	0.21	0.5 (-0.3, 1.3)	0.26	-0.3 (-1.3, 0.7)	0.54	5.1 (4.4, 5.9)	< 0.0001
**ratioBDNF (pg/ml)**	lin.	0.2 (-0.2, 0.5)	0.31	0.1 (-0.2, 0.4)	0.43	-0.1 (-0.5, 0.3)	0.51	-9.1 (-9.3, -8.8)	< 0.0001

The column reg. defines the type of multilevel model (mlm) used for the outcome; lin. = linear, log lin. = linear mlm on logged values, binom. = logistic, poiss. = poisson, beta = mlm based on beta regression. Mini-BESTest = the Mini-Balance Evaluation Systems Test, Frändin-Grimby = the Frändin-Grimby scale, MDS-UPDRS = Movement Disorders Society - Unified Parkinson's Disease Rating Scale, ABC = the Activities-specific Balance Confidence Scale, mBDNF = mature BDNF, PDQ-39 = the Parkinson's Disease Questionnaire-39, EQ5D- VAS = the EuroQol 5 dimensions visual analogue scale, HADS = the Hospital Anxiety and Depression scale, proBDNF = precursor BDNF, ratioBDNF = proBDNF/mBDNF.

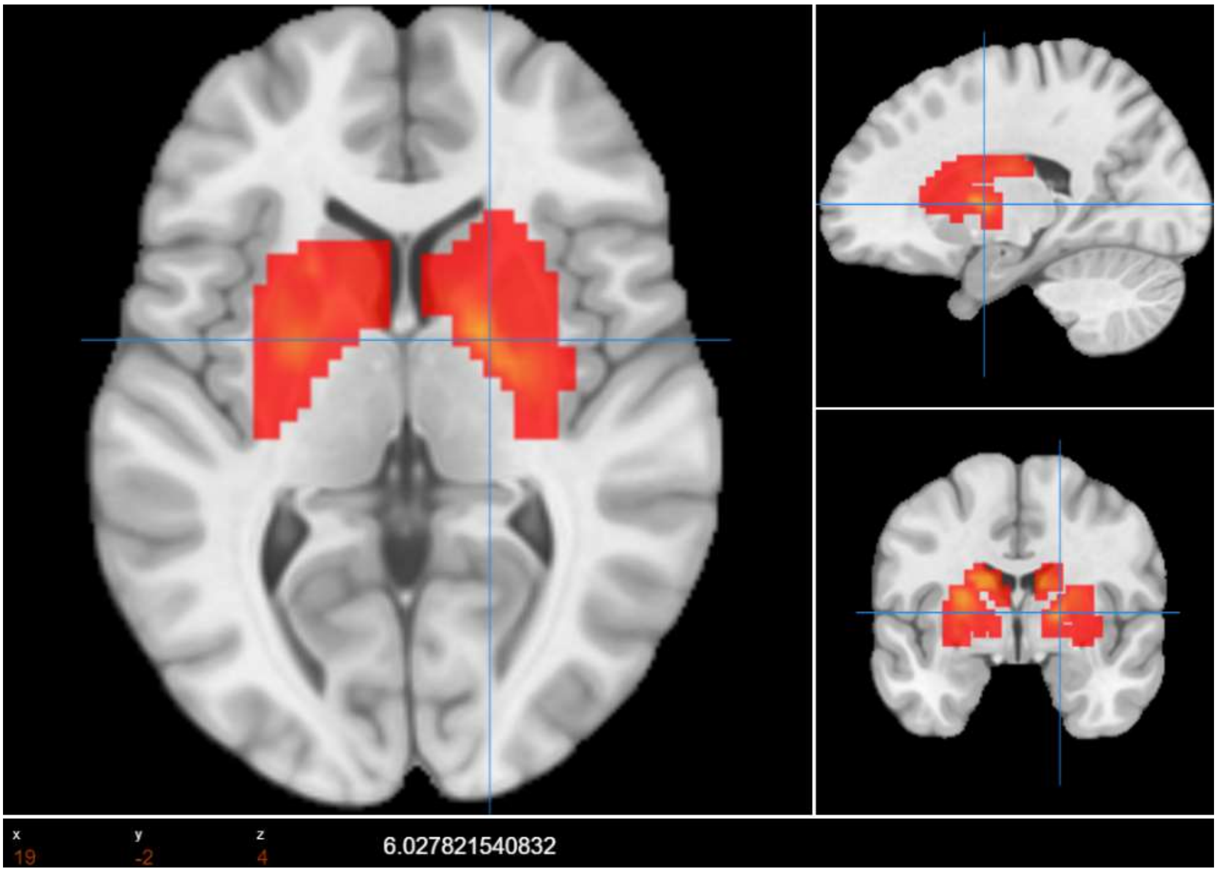

Supplementary Figure 1: The f-values map of the striatum from the time by group interaction analysis. A more yellow colour indicates a higher f-value, range: 0 (deepest red) to 8.9 (brightest yellow). Contrast: sequence trial blocks/random trial blocks. An interactive whole brain f-values map from the time by group interaction analysis can be found here: https://neurovault.org/collections/DORLUDYE/

Supplementary Table 6. Difference score correlations within the groups between the behavioural outcomes and the fMRI data and mBDNF.

		Mini-BESTest			Gait speed			Executive function		
	**group**	**rho**	**p**	**adj. p**	**rho**	**p**	**adj. p**	**rho**	**p**	**adj. p**
**Striatum**	HiBalance	-0.06	0.75	0.97	-0.10	0.58	0.97	-0.09	0.64	0.97
	Active control	0.13	0.50	0.97	0.24	0.22	0.97	-0.03	0.89	0.97
**Primary**	HiBalance	0.02	0.91	0.97	0.06	0.75	0.97	0.21	0.26	0.97
**motor c.**	Active control	-0.11	0.57	0.97	0.19	0.34	0.97	0.03	0.87	0.97
**Premotor c.**	HiBalance	0.06	0.75	0.97	0.14	0.45	0.97	0.10	0.61	0.97
	Active control	0.04	0.83	0.97	0.26	0.18	0.97	0.00	0.99	0.99
**Suppl**	HiBalance	0.03	0.87	0.97	0.05	0.77	0.97	-0.07	0.71	0.97
**motor c.**	Active control	-0.02	0.91	0.97	0.24	0.21	0.97	0.11	0.58	0.97
**ACC**	HiBalance	0.16	0.41	0.97	-0.2	0.28	0.97	-0.03	0.88	0.97
	Active control	-0.16	0.56	0.97	0.46	0.02	0.54	-0.15	0.47	0.97
**DLPFC**	HiBalance	0.18	0.33	0.97	-0.15	0.41	0.97	0.09	0.63	0.97
	Active control	0.07	0.71	0.97	0.29	0.14	0.97	-0.01	0.97	0.99
**mBDNF**	HiBalance	0.03	0.86	0.86	0.17	0.39	0.86	0.09	0.64	0.86
	Active control	0.05	0.83	0.86	-0.20	0.37	0.86	-0.07	0.76	0.86

rho = Spearman's rank order correlation coefficients. adj. p = false discovery rate adjusted p (alpha = 0.05). Primary motor c. = Primary motor cortex, Suppl. motor c. = Supplementary motor cortex, ACC = anterior cingulate cortex, DLPFC = dorsolateral prefrontal cortex, mBDNF = mature BDNF.

Supplementary Table 7. Comparison of the difference score correlations between the behavioural outcomes and brain activity and mBDNF, over the groups.

	Mini-BESTest			Gait speed			Executive function		
	**z**	**p**	**adj. p**	**z**	**p**	**adj. p**	**z**	**p**	**adj. p**
**Striatum**	0.72	0.47	0.71	1.29	0.20	0.59	0.21	0.83	0.83
**Primary motor c.**	0.49	0.63	0.84	0.48	0.63	0.84	0.65	0.51	0.84
**Premotor c.**	0.06	0.95	0.95	0.47	0.63	0.84	0.34	0.73	0.84
**Suppl. Motor c.**	0.20	0.85	0.91	0.72	0.47	0.84	0.65	0.51	0.84
**DLPFC**	0.40	0.69	0.84	1.67	0.10	0.72	0.36	0.72	0.84
**ACC**	0.99	0.32	0.84	2.70	0.010	0.10	0.43	0.66	0.84
**mBDNF**	0.05	0.96	1.0	1.25	1.0	1.0	0.53	0.59	1.0

z = estimates of the Fischer's Z significance tests**.** adj. p = false discovery rate adjusted p (alpha = 0.05). Primary motor c. = Primary motor cortex, Suppl. motor c. = Supplementary motor cortex, ACC = anterior cingulate cortex, DLPFC = dorsolateral prefrontal cortex, mBDNF = mature BDNF.

Supplementary Table 8. Estimates of the MLM analysis of the reaction time outcome of the serial reaction time task (motor learning task performed during fMRI).																	
**Seq.**		**Group**		**Time**		**Seq. x Time x Group**		**Seq. x Group**		**Seq. x time**		**Group x time**		**Trial order**		**Intercept**	
**b (ci)**	**p**	**b (ci)**	**p**	**b (ci)**	**p**	**b (ci)**	**p**	**b (ci)**	**p**	**b (ci)**	**p**	**b (ci)**	**p**	**b (ci)**	**p**	**b (ci)**	**p**
-0.01 (-0, -0)	0.020	-0.01 (-0, 0)	0.63	-0.03 (0, 0)	< 0.0001	0 (0, 0)	0.53	0.01 (-0, 0)	0.23	-0.01 (-0, -0)	0.00030	0.01 (0, 0)	0.035	0 (0, 0)	< 0.0001	0.63 (0.6, 0.7)	< 0.0001

Seq. = type of block (hidden sequence or random trials), b = unstandardised estimates.

Supplementary Table 9. Estimates of the MLM per protocol analyses.

	reg	Group b (95% CI)	Group p	Time b (95% CI)	Time p	Group x Time b (95% CI)	Group x Time p	Intercept b (95% CI)	Intercept p
**Mini-BESTest**	lin.	-0.3 (-1.8, 1.1)	0.68	0.4 (-0.4, 1.2)	0.30	0.5 (-0.6, 1.5)	0.37	21.4 (20.4, 22.5)	< 0.0001
**Gait speed (m/sec)**	lin.	0.002 (-0.08, 0.09)	0.95	0.01 (-0.03, 0.05)	0.58	0.05 (-0.002, 0.1)	0.061	1.2 (1.2, 1.3)	< 0.0001
**Step length**	lin.	0.6 (-3.4, 4.5)	0.78	0.6 (-0.9, 2.1)	0.45	1.3 (-0.8, 3.4)	0.22	65.6 (62.7, 68.5)	< 0.0001
**Walk 12**	poiss.	0.1 (-0.3, 0.5)	0.50	0 (-0.1, 0.2)	0.90	-0.1 (-0.3, 0.1)	0.27	2 (1.7, 2.3)	< 0.0001
**Steps/day**	log lin.	-0.1 (-0.4, 0.2)	0.64	0 (-0.1, 0.1)	0.63	-0.1 (-0.2, 0.1)	0.31	8.6 (8.4, 8.8)	< 0.0001
**Frändin -Grimby**	binom.	0 (-1.4, 1.3)	0.96	-0.6 (-1.9, 0.6)	0.30	1 (-0.6, 2.7)	0.23	-0.4 (-1.5, 0.6)	0.39
**MDS-UPDRS III**	lin.	1.2 (-4, 6.5)	0.64	-1.7 (-5.1, 1.8)	0.35	-0.9 (-5.7, 3.8)	0.70	30.4 (26.6, 34.2)	< 0.0001
**MDS-UPDRS Tot.**	lin.	2.2 (-5.8, 10.3)	0.59	-3.5 (-7.7, 0.7)	0.11	0 (-5.8, 5.8)	0.99	49.3 (43.4, 55.2)	< 0.0001
**ABC**	beta	-0.1 (-0.7, 0.5)	0.68	0 (-0.2, 0.3)	0.66	0.2 (-0.1, 0.4)	0.26	1.9 (1.5, 2.3)	< 0.0001
**Executive functions**	lin.	1.1 (-0.1, 2.3)	0.073	0.6 (0.2, 1.1)	0.011	0.2 (-0.5, 0.8)	0.62	-0.5 (-1.4, 0.4)	0.26
**mBDNF**	lin.	293.5 (-2779, 3366)	0.85	-276.5 (-2103.5, 1550.5)	0.77	238.3 (-2241.8, 2718.5)	0.85	36678.1 (34434.3, 38922)	< 0.0001
**Voice int**	lin.	-0.2 (-2, 1.6)	0.79	2 (1.1, 2.9)	0.0001	-2.2 (-3.5, -0.9)	0.0012	71 (69.7, 72.3)	< 0.0001
**PDQ-39**	log. lin.	0.2 (-0.2, 0.6)	0.29	-0.2 (-0.3, 0)	0.053	-0.1 (-0.3, 0.2)	0.58	2.7 (2.4, 3)	< 0.0001
**EQ5D VAS**	beta	-0.4 (-0.9, 0.1)	0.082	0 (-0.2, 0.3)	0.75	0.2 (-0.2, 0.5)	0.27	1.3 (1, 1.7)	< 0.0001
**HADS dep.**	poiss. -	0 (-0.4, 0.5)	0.85	0 (-0.3, 0.2)	0.80	0 (-0.3, 0.4)	0.94	0.9 (0.6, 1.3)	< 0.0001
**HADS anx.**	poiss. -	0.2 (-0.2, 0.6)	0.28	0 (-0.2, 0.3)	0.69	-0.1 (-0.4, 0.2)	0.47	1.1 (0.8, 1.4)	< 0.0001
**proBDNF (pg/ml)**	lin.	0.2 (-1, 1.4)	0.76	0.3 (0, 0.5)	0.054	-0.3 (-0.7, 0)	0.085	5.7 (4.8, 6.6)	< 0.0001
**ratioBDNF (pg/ml)**	lin.	0 (-1.2, 1.3)	0.95	0.2 (0, 0.5)	0.058	-0.3 (-0.6, 0)	0.10	-4.8 (-5.8, -3.9)	< 0.0001

The column reg. defines the type of multilevel model (mlm) used for the outcome; lin. = linear, log lin. = linear mlm on logged values, binom. = logistic, poiss. = poisson, beta = mlm based on beta regression. Mini-BESTest = the Mini-Balance Evaluation Systems Test, Frändin-Grimby = the Frändin-Grimby scale, MDS-UPDRS = Movement Disorders Society - Unified Parkinson's Disease Rating Scale, ABC = the Activities-specific Balance Confidence Scale, mBDNF = mature BDNF, PDQ-39 = the Parkinson's Disease Questionnaire-39, EQ5D- VAS = the EuroQol 5 dimensions visual analogue scale, HADS = the Hospital Anxiety and Depression scale, proBDNF = precursor BDNF, ratioBDNF = proBDNF/mBDNF.

Supplementary References

1 Franchignoni, F., Horak, F., Godi, M., Nardone, A. & Giordano, A. Using psychometric techniques to improve the Balance Evaluation Systems Test: the mini-BESTest. *J. Rehabil. Med.* **42**, 323-331, doi:10.2340/16501977-0537 (2010).

2 Lofgren, N., Lenholm, E., Conradsson, D., Stahle, A. & Franzen, E. The Mini-BESTest-a clinically reproducible tool for balance evaluations in mild to moderate Parkinson's disease? *BMC Neurol.* **14**, 235, doi:10.1186/s12883-014-0235-7 (2014).

3 Bladh, S. *et al.* Psychometric performance of a generic walking scale (Walk-12G) in multiple sclerosis and Parkinson's disease. *J. Neurol.* **259**, 729-738, doi:10.1007/s00415-011-6254-z (2012).

4 Grimby, G. & Frandin, K. On the use of a six-level scale for physical activity. *Scand. J. Med. Sci. Sports* **28**, 819-825, doi:10.1111/sms.12991 (2018).

5 Goetz, C. G. *et al.* Movement Disorder Society-sponsored revision of the Unified Parkinson's Disease Rating Scale (MDS-UPDRS): scale presentation and clinimetric testing results. *Mov. Disord.* **23**, 2129-2170, doi:10.1002/mds.22340 (2008).

6 Hoehn, M. M. & Yahr, M. D. Parkinsonism: onset, progression and mortality. *Neurology* **17**, 427-442, doi:10.1212/wnl.17.5.427 (1967).

7 Powell, L. E. & Myers, A. M. The Activities-specific Balance Confidence (ABC) Scale. *J. Gerontol. A Biol. Sci. Med. Sci.* **50A**, M28-34, doi:10.1093/gerona/50a.1.m28 (1995).

8 Weschler, D. *Wechsler Adult Intelligence Scale - Fourth Edition*. (Stat Solut, 2008).

9 Delis, D. C., Kaplan, E. & Kramer, J. H. *Delis-Kaplan executive function system: Examiner's manual.*, (The Psychological Corporation, 2001).

10 Jenkinson, C., Fitzpatrick, R., Peto, V., Greenhall, R. & Hyman, N. The Parkinson's Disease Questionnaire (PDQ-39): development and validation of a Parkinson's disease summary index score. *Age Ageing* **26**, 353-357, doi:10.1093/ageing/26.5.353 (1997).

11 EuroQolGroup. EuroQol - a new facility for the measurement of health-related quality of life. *Health Policy* **16**, 199-208, doi:10.1016/0168-8510(90)90421-9 (1990).

12 Rodriguez-Blazquez, C. *et al.* Psychometric attributes of the Hospital Anxiety and Depression Scale in Parkinson's disease. *Mov. Disord.* **24**, 519-525, doi:10.1002/mds.22321 (2009).

13 Nissen, M. J. & Bullemer, P. Attentional Requirements of Learning - Evidence from Performance-Measures. *Cogn. Psychol.* **19**, 1-32, doi:10.1016/0010-0285(87)90002-8 (1987).

14 Freidle, M. *et al.* Measuring implicit sequence learning and dual task ability in mild to moderate Parkinson s disease: A feasibility study. *PLoS One* **16**, e0251849, doi:10.1371/journal.pone.0251849 (2021).

15 Borkovec, T. D. & Nau, S. D. Credibility of analogue therapy rationales. *J. Behav. Ther. Exp. Psychiatry* **3**, 257-260, doi:10.1016/0005-7916(72)90045-6 (1972).

16 R: A Language and Environment for Statistical Computing (R Foundation for Statistical Computing, Vienna, Austria, 2020).
