# Supplementary material for: Behavioural and neuroplastic effects of a double-blind randomised controlled balance exercise trial in people with Parkinson’s disease
Source: NPJ Parkinsons Dis. 2022 Jan 21;8:12. doi: 10.1038/s41531-021-00269-5 (PMC8782921; doi:10.1038/s41531-021-00269-5)
Supplement: Supplementary file 2 — Reporting Summary [file 41531_2021_269_MOESM2_ESM.pdf]

nature portfolio

Corresponding author(s): Malin Freidle

Last updated by author(s): Nov 24, 2021

Reporting Summary

Nature Portfolio wishes to improve the reproducibility of the work that we publish. This form provides structure for consistency and transparency in reporting. For further information on Nature Portfolio policies, see our Editorial Policies and the Editorial Policy Checklist.

Statistics

For all statistical analyses, confirm that the following items are present in the figure legend, table legend, main text, or Methods section.

n/a	Confirmed	
☐	☒	The exact sample size (*n*) for each experimental group/condition, given as a discrete number and unit of measurement
☐	☒	A statement on whether measurements were taken from distinct samples or whether the same sample was measured repeatedly
☐	☒	The statistical test(s) used AND whether they are one- or two-sided<br>*Only common tests should be described solely by name; describe more complex techniques in the Methods section.*
☐	☒	A description of all covariates tested
☐	☒	A description of any assumptions or corrections, such as tests of normality and adjustment for multiple comparisons
☐	☒	A full description of the statistical parameters including central tendency (e.g. means) or other basic estimates (e.g. regression coefficient) AND variation (e.g. standard deviation) or associated estimates of uncertainty (e.g. confidence intervals)
☐	☒	For null hypothesis testing, the test statistic (e.g. *F*, *t*, *r*) with confidence intervals, effect sizes, degrees of freedom and *P* value noted<br>*Give P values as exact values whenever suitable.*
☒	☐	For Bayesian analysis, information on the choice of priors and Markov chain Monte Carlo settings
☐	☒	For hierarchical and complex designs, identification of the appropriate level for tests and full reporting of outcomes
☐	☒	Estimates of effect sizes (e.g. Cohen's *d*, Pearson's *r*), indicating how they were calculated

Our web collection on statistics for biologists contains articles on many of the points above.

Software and code

Policy information about availability of computer code

Data collection	Gait speed and step length were assessed using an electronic walkway (GAITRite®, CIR Systems, Inc., Havertown, PA, USA)<br>Habitual physical activity (steps per day) was measured by an accelerometer (Actigraph GT3X+, Pensacola, FL, USA)<br>Voice sound level was recorded using the equipment Sony Digital Audio Tape Deck DTC-ZE700 and the software Sopran (version 1.0.22 © Tolvan Data)<br>Indirect measures of brain activity were acquired by fMRI usign a 3T Phillips Ingenia scanner with a 15channel head coil.<br>The serial reaction time task was presented to the participants inside the scanner using the software Psychopy (version 1.85.4).
Data analysis	We used R 4.0.3. for the multiple imputation, the statistical group analyses of the behavioural outcomes and BDNF outcomes as well as for the difference score correlations. Initial quality control of MRI data was done using MRIQC and the preprocessing was done using fMRIPrep. We used SPM12 (version 7771) for the first and second level analyses of the fMRI data. The ELISA kit was used for prestatistical handling and analyses of the blood serum sample BDNF.

For manuscripts utilizing custom algorithms or software that are central to the research but not yet described in published literature, software must be made available to editors and reviewers. We strongly encourage code deposition in a community repository (e.g. GitHub). See the Nature Portfolio guidelines for submitting code & software for further information.

Data

Policy information about availability of data

All manuscripts must include a data availability statement. This statement should provide the following information, where applicable:

- Accession codes, unique identifiers, or web links for publicly available datasets
- A description of any restrictions on data availability
- For clinical datasets or third party data, please ensure that the statement adheres to our policy

With respect to the Swedish and EU personal data legislation (GDPR), the data is not freely accessible due to regulations regarding personal integrity in research, public access, and privacy. The data is available from the principal investigator of the project: Erika Franzén (erika.franzen@ki.se), on reasonable request. Any sharing of data will be regulated via a data transfer and user agreement with the recipient.

Field-specific reporting

Please select the one below that is the best fit for your research. If you are not sure, read the appropriate sections before making your selection.

☒ Life sciences ☐ Behavioural & social sciences ☐ Ecological, evolutionary & environmental sciences

For a reference copy of the document with all sections, see nature.com/documents/nr-reporting-summary-flat.pdf

Life sciences study design

All studies must disclose on these points even when the disclosure is negative.

Sample size	An independent statistician performed a power calculation using 2000 bootstrap samples and the variance estimates from our pilot study. By testing a random-intercept model with group, time, and their interaction as covariates and the alpha level set to 0.05 (two-sided), it was estimated that a sample size of 40 individuals per group would result in a power of 82% to detect a between-group difference of two points in the mean of the total score of the Mini-BESTest at post assessment. The two point difference was based on the effect of similar intervention studies and the measurement error of the Mini-BESTest. To account for dropouts and data exclusion due to technical problems or low imaging quality, we aimed for 50 participants in each group.
Data exclusions	Based on quality control of the MRI data using MRIQC and the output of the preprocessing (fMRIPrep), we excluded three participants from the brain data analyses due the a mean framewise displacement greater than 0.5. Two participants lacked more than 80% of the voxels in the striatum (as defined by our atlas of the striatum) due to signal drop and were excluded from the analyses of striatal activity. These exclusion criteria were unfortunately not preregistered but are in line with the field's standard procedure and consensus and were decided on before any data analyses were performed.<br><br>We also performed complementary analyses of the behavioural outcomes and mBDNF, where solely participants who attended at least 60% of the training occasions were included.
Replication	Two main things were done to increase the reproducibility of our findings;<br>1) We made a detailed analyses plan describing the hypotheses, their rankings and the analyses to be made and thereby reduced the risk of bias.<br>2) All preparation and cleaning of our data as well as all analyses and plots were made using scripts.<br>The analyses plan and all scripts for the analyses as well as the program files to run the motor task used during scanning can be found on our osf page https://osf.io/6txsk/
Randomization	For each consecutive wave, participants who met all eligibility criteria were randomly allocated (1:1) to the HiBalance program or the active control group. The randomisation was based on a true random number service (http://www. random.org) and performed by an individual not responsible for assessment or data analysis. The participants were informed of their group allocation through sealed opaque envelopes.
Blinding	All assessors were blinded to group allocation, and participants were instructed not to disclose any information of their program content during the post-intervention assessments. The assessors reported their perceived level of blinding after each assessment by use of a questionnaire with results showing a successful blinding, see result section and supplement. The blinding was kept throughout the statistical analyses using arbitrary group indicators.

Behavioural & social sciences study design

All studies must disclose on these points even when the disclosure is negative.

Study description	*Briefly describe the study type including whether data are quantitative, qualitative, or mixed-methods (e.g. qualitative cross-sectional, quantitative experimental, mixed-methods case study).*
Research sample	*State the research sample (e.g. Harvard university undergraduates, villagers in rural India) and provide relevant demographic information (e.g. age, sex) and indicate whether the sample is representative. Provide a rationale for the study sample chosen. For studies involving existing datasets, please describe the dataset and source.*

Sampling strategy	*Describe the sampling procedure (e.g. random, snowball, stratified, convenience). Describe the statistical methods that were used to predetermine sample size OR if no sample-size calculation was performed, describe how sample sizes were chosen and provide a rationale for why these sample sizes are sufficient. For qualitative data, please indicate whether data saturation was considered, and what criteria were used to decide that no further sampling was needed.*
Data collection	*Provide details about the data collection procedure, including the instruments or devices used to record the data (e.g. pen and paper, computer, eye tracker, video or audio equipment) whether anyone was present besides the participant(s) and the researcher, and whether the researcher was blind to experimental condition and/or the study hypothesis during data collection.*
Timing	*Indicate the start and stop dates of data collection. If there is a gap between collection periods, state the dates for each sample cohort.*
Data exclusions	*If no data were excluded from the analyses, state so OR if data were excluded, provide the exact number of exclusions and the rationale behind them, indicating whether exclusion criteria were pre-established.*
Non-participation	*State how many participants dropped out/declined participation and the reason(s) given OR provide response rate OR state that no participants dropped out/declined participation.*
Randomization	*If participants were not allocated into experimental groups, state so OR describe how participants were allocated to groups, and if allocation was not random, describe how covariates were controlled.*

Ecological, evolutionary & environmental sciences study design

All studies must disclose on these points even when the disclosure is negative.

Study description	*Briefly describe the study. For quantitative data include treatment factors and interactions, design structure (e.g. factorial, nested, hierarchical), nature and number of experimental units and replicates.*
Research sample	*Describe the research sample (e.g. a group of tagged Passer domesticus, all Stenocereus thurberi within Organ Pipe Cactus National Monument), and provide a rationale for the sample choice. When relevant, describe the organism taxa, source, sex, age range and any manipulations. State what population the sample is meant to represent when applicable. For studies involving existing datasets, describe the data and its source.*
Sampling strategy	*Note the sampling procedure. Describe the statistical methods that were used to predetermine sample size OR if no sample-size calculation was performed, describe how sample sizes were chosen and provide a rationale for why these sample sizes are sufficient.*
Data collection	*Describe the data collection procedure, including who recorded the data and how.*
Timing and spatial scale	*Indicate the start and stop dates of data collection, noting the frequency and periodicity of sampling and providing a rationale for these choices. If there is a gap between collection periods, state the dates for each sample cohort. Specify the spatial scale from which the data are taken*
Data exclusions	*If no data were excluded from the analyses, state so OR if data were excluded, describe the exclusions and the rationale behind them, indicating whether exclusion criteria were pre-established.*
Reproducibility	*Describe the measures taken to verify the reproducibility of experimental findings. For each experiment, note whether any attempts to repeat the experiment failed OR state that all attempts to repeat the experiment were successful.*
Randomization	*Describe how samples/organisms/participants were allocated into groups. If allocation was not random, describe how covariates were controlled. If this is not relevant to your study, explain why.*
Blinding	*Describe the extent of blinding used during data acquisition and analysis. If blinding was not possible, describe why OR explain why blinding was not relevant to your study.*

Did the study involve field work? ☐ Yes ☐ No

Field work, collection and transport

Field conditions	*Describe the study conditions for field work, providing relevant parameters (e.g. temperature, rainfall).*
Location	*State the location of the sampling or experiment, providing relevant parameters (e.g. latitude and longitude, elevation, water depth).*
Access & import/export	*Describe the efforts you have made to access habitats and to collect and import/export your samples in a responsible manner and in compliance with local, national and international laws, noting any permits that were obtained (give the name of the issuing authority, the date of issue, and any identifying information).*
Disturbance	*Describe any disturbance caused by the study and how it was minimized.*

Reporting for specific materials, systems and methods

We require information from authors about some types of materials, experimental systems and methods used in many studies. Here, indicate whether each material, system or method listed is relevant to your study. If you are not sure if a list item applies to your research, read the appropriate section before selecting a response.

Materials & experimental systems

n/a	Involved in the study
☒	☐ Antibodies
☒	☐ Eukaryotic cell lines
☒	☐ Palaeontology and archaeology
☒	☐ Animals and other organisms
☐	☒ Human research participants
☐	☒ Clinical data
☒	☐ Dual use research of concern

Methods

n/a	Involved in the study
☒	☐ ChIP-seq
☒	☐ Flow cytometry
☐	☒ MRI-based neuroimaging

Antibodies

Antibodies used

Describe all antibodies used in the study; as applicable, provide supplier name, catalog number, clone name, and lot number.

Validation

Describe the validation of each primary antibody for the species and application, noting any validation statements on the manufacturer's website, relevant citations, antibody profiles in online databases, or data provided in the manuscript.

Eukaryotic cell lines

Policy information about cell lines

Cell line source(s)

State the source of each cell line used.

Authentication

Describe the authentication procedures for each cell line used OR declare that none of the cell lines used were authenticated.

Mycoplasma contamination

Confirm that all cell lines tested negative for mycoplasma contamination OR describe the results of the testing for mycoplasma contamination OR declare that the cell lines were not tested for mycoplasma contamination.

Commonly misidentified lines
(See ICLAC register)

Name any commonly misidentified cell lines used in the study and provide a rationale for their use.

Palaeontology and Archaeology

Specimen provenance

Provide provenance information for specimens and describe permits that were obtained for the work (including the name of the issuing authority, the date of issue, and any identifying information). Permits should encompass collection and, where applicable, export.

Specimen deposition

Indicate where the specimens have been deposited to permit free access by other researchers.

Dating methods

If new dates are provided, describe how they were obtained (e.g. collection, storage, sample pretreatment and measurement), where they were obtained (i.e. lab name), the calibration program and the protocol for quality assurance OR state that no new dates are provided.

☐ Tick this box to confirm that the raw and calibrated dates are available in the paper or in Supplementary Information.

Ethics oversight

Identify the organization(s) that approved or provided guidance on the study protocol, OR state that no ethical approval or guidance was required and explain why not.

Note that full information on the approval of the study protocol must also be provided in the manuscript.

Animals and other organisms

Policy information about studies involving animals; ARRIVE guidelines recommended for reporting animal research

Laboratory animals

For laboratory animals, report species, strain, sex and age OR state that the study did not involve laboratory animals.

Wild animals

Provide details on animals observed in or captured in the field; report species, sex and age where possible. Describe how animals were caught and transported and what happened to captive animals after the study (if killed, explain why and describe method; if released, say where and when) OR state that the study did not involve wild animals.

Field-collected samples	*For laboratory work with field-collected samples, describe all relevant parameters such as housing, maintenance, temperature, photoperiod and end-of-experiment protocol OR state that the study did not involve samples collected from the field.*
Ethics oversight	*Identify the organization(s) that approved or provided guidance on the study protocol, OR state that no ethical approval or guidance was required and explain why not.*

Note that full information on the approval of the study protocol must also be provided in the manuscript.

Human research participants

Policy information about studies involving human research participants

Population characteristics	Included participants had mild to moderate idiopathic PD with Hoehn and Yahr stage 2 (n = 73) or 3 (n = 22), they were ≥ 60 years of age (mean = 71 years), 35 were women and 60 men. Participants were excluded if they had any other disorder that substantially influenced balance, voice- or speech performance.
Recruitment	Participants were recruited in four successive waves from 2018 to 2019 via advertisements in local newspapers and through the Swedish Parkinson Association. Following an initial telephone screening, eligibility was established at an in-person assessment in a university setting.<br><br>There might be a possible bias in that only individuals willing to participate in an extensive study with several assessments and commitment to participate in the training programs, applied for participation. This may to some extent limit the generalizibility of our results.
Ethics oversight	The Regional Ethical Review Board in Stockholm (2016/1264–31/4, 2017/1258– 32 and 2017/2445–32).

Note that full information on the approval of the study protocol must also be provided in the manuscript.

Clinical data

Policy information about clinical studies

All manuscripts should comply with the ICMJE guidelines for publication of clinical research and a completed CONSORT checklist must be included with all submissions.

Clinical trial registration	NCT03213873
Study protocol	Published study protocol: https://pubmed.ncbi.nlm.nih.gov/31718583/<br>Preregistered detailed analysis plan: https://osf.io/6txsk/
Data collection	Inclusion of participants occurred between Jan 15, 2018 and Sep 9, 2019. After telephone screening, participants were invited to first-person assessments in our movement lab at Karolinska Institute. For included participants this assessment was followed by one session of brain imaging with MRI, one session with assessments of cognitive functions and speech- and voice function, and blood sampling, all performed in a university hospital setting. The pre and post assessments were done 1-3 weeks before and after the training programs.
Outcomes	The primary outcome was balance performance assessed with the Mini-BESTest, a rating scale for dynamic balance validated in people with PD.<br><br>The secondary behavioural outcomes included comfortable gait speed and step length assessed on an electronic walkway (GAITRite®, CIR Systems, Inc., Havertown, PA, USA), and self-reported gait ability (the Walk 12 scale). Habitual physical activity (steps per day) was measured by an accelerometer for seven consecutive days, and self-reported level of physical activity through the Frändin-Grimby scale. Various motor and non-motor aspects of PD were captured using the total score on the Movement Disorder Society – Unified Parkinson's Disease Rating Scale (MDS-UPDRS), whereas motor function specifically was addressed through part III of the same scale. Balance confidence was reported using the Activities-specific Balance Confidence scale (ABC scale). Executive function was assessed with a composite measure of three tests from the Delis-Kaplan Executive Function System (letter fluency and category switching from Verbal Fluency, and the switch condition from the Color-Word Interference Test), and one test measure from the Wechsler Adult Intelligence Scale (Digit Span total score). Recordings of speech and voice were used to investigate the effects of the HiCommunication training. The recordings were performed according to standardised routines for high-quality recordings in a sound-proof recording studio with the equipment Sony Digital Audio Tape Deck DTC-ZE700 and the software Sopran (version 1.0.22 © Tolvan Data). The outcome measure from the studio recordings used in the present study, was mean voice sound level (dB SPL) in reading a Swedish standardised text. Self-reported data on health-related quality of life was collected using Parkinson's Disease Questionnaire-39 (PDQ-39) and EuroQol-5 Dimensions-VAS (EQ-5D VAS) and symptoms of depression and anxiety were measured with the Hospital Anxiety and Depression scale (HADS).<br><br>Indirect measures of brain activity were acquired by fMRI and the blood-oxygen-level-dependent (BOLD) signal during performance of a computer-based motor learning task named the serial reaction time task.<br><br>Blood samples were collected before and after the interventions to analyse serum-levels of BDNF, primarily mature BDNF (mBDNF).

Dual use research of concern

Policy information about dual use research of concern

Hazards

Could the accidental, deliberate or reckless misuse of agents or technologies generated in the work, or the application of information presented in the manuscript, pose a threat to:

No	Yes	
☐	☐	Public health
☐	☐	National security
☐	☐	Crops and/or livestock
☐	☐	Ecosystems
☐	☐	Any other significant area

Experiments of concern

Does the work involve any of these experiments of concern:

No	Yes	
☐	☐	Demonstrate how to render a vaccine ineffective
☐	☐	Confer resistance to therapeutically useful antibiotics or antiviral agents
☐	☐	Enhance the virulence of a pathogen or render a nonpathogen virulent
☐	☐	Increase transmissibility of a pathogen
☐	☐	Alter the host range of a pathogen
☐	☐	Enable evasion of diagnostic/detection modalities
☐	☐	Enable the weaponization of a biological agent or toxin
☐	☐	Any other potentially harmful combination of experiments and agents

ChIP-seq

Data deposition

☐ Confirm that both raw and final processed data have been deposited in a public database such as GEO.

☐ Confirm that you have deposited or provided access to graph files (e.g. BED files) for the called peaks.

Data access links<br>*May remain private before publication.*	*For "Initial submission" or "Revised version" documents, provide reviewer access links. For your "Final submission" document, provide a link to the deposited data.*
Files in database submission	*Provide a list of all files available in the database submission.*
Genome browser session<br>(e.g. UCSC)	*Provide a link to an anonymized genome browser session for "Initial submission" and "Revised version" documents only, to enable peer review. Write "no longer applicable" for "Final submission" documents.*

Methodology

Replicates	*Describe the experimental replicates, specifying number, type and replicate agreement.*
Sequencing depth	*Describe the sequencing depth for each experiment, providing the total number of reads, uniquely mapped reads, length of reads and whether they were paired- or single-end.*
Antibodies	*Describe the antibodies used for the ChIP-seq experiments; as applicable, provide supplier name, catalog number, clone name, and lot number.*
Peak calling parameters	*Specify the command line program and parameters used for read mapping and peak calling, including the ChIP, control and index files used.*
Data quality	*Describe the methods used to ensure data quality in full detail, including how many peaks are at FDR 5% and above 5-fold enrichment.*
Software	*Describe the software used to collect and analyze the ChIP-seq data. For custom code that has been deposited into a community repository, provide accession details.*

Flow Cytometry

Plots

Confirm that:

- [ ] The axis labels state the marker and fluorochrome used (e.g. CD4-FITC).
- [ ] The axis scales are clearly visible. Include numbers along axes only for bottom left plot of group (a 'group' is an analysis of identical markers).
- [ ] All plots are contour plots with outliers or pseudocolor plots.
- [ ] A numerical value for number of cells or percentage (with statistics) is provided.

Methodology

Sample preparation	*Describe the sample preparation, detailing the biological source of the cells and any tissue processing steps used.*
Instrument	*Identify the instrument used for data collection, specifying make and model number.*
Software	*Describe the software used to collect and analyze the flow cytometry data. For custom code that has been deposited into a community repository, provide accession details.*
Cell population abundance	*Describe the abundance of the relevant cell populations within post-sort fractions, providing details on the purity of the samples and how it was determined.*
Gating strategy	*Describe the gating strategy used for all relevant experiments, specifying the preliminary FSC/SSC gates of the starting cell population, indicating where boundaries between "positive" and "negative" staining cell populations are defined.*

- [ ] Tick this box to confirm that a figure exemplifying the gating strategy is provided in the Supplementary Information.

Magnetic resonance imaging

Experimental design

Design type	Task-fMRI with block design
Design specifications	The serial reaction time task performed in the scanner was 9 minutes long and consisted of 10 blocks of trials, each block interleaved by a six second break. Each block consisted of 40 trials with a duration of 1.2 seconds. Unbeknownst to the participants, in 6 of the 10 blocks, the trials followed a 10–item higher order sequence. Two different sequences but with the same characteristics were used for the pre and post assessment, respectively.
Behavioral performance measures	Accuracy of button presses and response time were recorded. The statistical analyses was made on the response time as this is the standard outcome used for the serial reaction time task. Accuracy was however used to exclude incorrect button presses/trials from the analyses of response time.<br><br>Before performing the serial reaction time task in the scanner, the participants practiced the task seated at a table outside the scanner room, using the same type of response pads as used inside the scanner. An experiment leader helped the participants understand the task and how to use the response pads provided to make sure all participants would know how to perform the task correctly inside the scanner. The training ended when the participant achieved 80% accuracy (after at least two rounds of training) or after a maximum of five rounds. Each round included 40 trials/ button presses.

Acquisition

Imaging type(s)	Functional
Field strength	3T
Sequence & imaging parameters	Echo-planar imaging (EPI) with gradient echo, repetition/echo time (TR/TE) = 2085/35ms, flip angle = 75°, voxel-size: 3.5×3.5×3.5mm, field of view: 224 x 224 x 140, 265 slices in ascending order, AcquisitionMatrixPE = 62.
Area of acquisition	Whole brain scan

Diffusion MRI ☐ Used ☒ Not used

Preprocessing

Preprocessing software	fMRIPrep 20.2.0 was used for all preprocessing steps except the smoothing which was done using SPM12 (7771) at 8 mm FWHM (default in SPM12).
Normalization	Normalization was done using the default settings of fMRIPrep 20.2.0. When available, the two T1 images from each

Normalization	individual's pre and post scan were merged and used as a longitudinal template for co-registration with the functional images. For individuals with field maps, these were included in the fMRIPrep pipeline.
Normalization template	MNI template 2009c.
Noise and artifact removal	To decrease noise and artifacts the 24 motion-derived regressors as well as the first five aCompCor regressors and the cosine regressors derived from the fMRIPrep preprocessing, were included as independent variables in the first-level analyses.
Volume censoring	No volume censoring was used.

Statistical modeling & inference

Model type and settings	Independent variables for first level analyses were the experimental timeline convoluted with the canonical hemodynamic function, 24 motion-derived regressors as well as the first five aCompCor regressors and the cosine regressors.<br><br>Group level analyses were performed using the flexible factorial model as implemented in SPM12.29. The group level analyses were performed separately for the striatum and for one mask comprising of multiple regions of interests that included the primary motor cortex, the premotor cortex, the supplementary motor cortex, the anterior cingulate cortex and the dorsolateral prefrontal cortex. A cluster-defining threshold of p = 0.05, family-wise error corrected, was used.<br><br>As for the difference score correlations, the mean of the 10% most active voxels (values larger during the sequence blocks than during the random blocks) was calculated for each ROI at pre and post to then enable calculation of delta values. Then, we calculated Spearman's rang order correlation coefficients within the two groups to estimate the correlations between the difference scores (pre – post assessment) of balance ability, gait speed and executive function with the difference scores of the activity in the ROIs (the striatum, the primary motor cortex, the premotor cortex, the supplementary motor cortex, the anterior cingulate cortex and the dorsolateral prefrontal cortex). We then used Fischer's significance test by first transforming the correlations coefficients to z-scores and then significance testing the transformed correlations coefficients over the two groups.
Effect(s) tested	Activity during random blocks of the serial reaction time task was contrasted to activity during the sequence blocks of the same task, creating statistical contrast maps for each individual and scan.<br>Group level analyses were performed using the flexible factorial model as implemented in SPM12.29.

Specify type of analysis: ☐ Whole brain ☐ ROI-based ☒ Both

Statistic type for inference (See Eklund et al. 2016)	Voxel-wise
Correction	Random field theory for small regions with alpha set to 0.05 was used for thresholding the statistical maps on the first level. Thresholding was done separately for statistical maps of striatum and for statistical maps of the remaining ROIs.<br><br>On group level, a cluster-defining threshold of p = 0.05, family-wise error corrected, was used.

Models & analysis

n/a	Involved in the study
☒	☐ Functional and/or effective connectivity
☒	☐ Graph analysis
☐	☒ Multivariate modeling or predictive analysis

Functional and/or effective connectivity	*Report the measures of dependence used and the model details (e.g. Pearson correlation, partial correlation, mutual information).*
Graph analysis	*Report the dependent variable and connectivity measure, specifying weighted graph or binarized graph, subject- or group-level, and the global and/or node summaries used (e.g. clustering coefficient, efficiency, etc.).*
Multivariate modeling and predictive analysis	Group level analyses were performed using the flexible factorial model as implemented in SPM12 with time and group and their interaction as independent variables.
